# Activation of automethylated PRC2 by dimerization on chromatin

**DOI:** 10.1016/j.molcel.2024.08.025

**Published:** 2024-09-19

**Authors:** Paul V. Sauer, Egor Pavlenko, Trinity Cookis, Linda C. Zirden, Juliane Renn, Ankush Singhal, Pascal Hunold, Michaela N. Hoehne-Wiechmann, Olivia van Ray, Farnusch Kaschani, Markus Kaiser, Robert Hänsel-Hertsch, Karissa Y. Sanbonmatsu, Eva Nogales, Simon Poepsel

**Affiliations:** 1 California Institute for Quantitative Biology (QB3), University of California, Berkeley, CA 94720, USA; 2 Howard Hughes Medical Institute, University of California, Berkeley, CA 94720, USA; 3 Center for Molecular Medicine Cologne (CMMC), Faculty of Medicine and University Hospital, University of Cologne, 50931 Cologne, Germany; 4 Department of Molecular and Cell Biology, University of California, Berkeley, CA 94720, USA; 5 Theoretical Biology and Biophysics, Theoretical Division, Los Alamos National Laboratory, Los Alamos, NM 87545, USA; 6 Department of Translational Genomics, Faculty of Medicine and University Hospital Cologne, University of Cologne, 50931 Cologne, Germany; 7 Department of Chemical Biology, University of Duisburg-Essen, Center for Medical Biotechnology (ZMB), Faculty of Biology, Essen, Germany; 8 Institute of Human Genetics, University Hospital Cologne, 50931 Cologne, Germany; 9 Cologne Excellence Cluster for Cellular Stress Responses in Ageing-Associated Diseases (CECAD), University of Cologne, 50931 Cologne, Germany; 10 Molecular Biophysics and Integrative Bio-Imaging Division, Lawrence Berkeley National Laboratory, Berkeley, CA 94720, USA; 11 These authors contributed equally; 12 Lead contact

## Abstract

Polycomb repressive complex 2 (PRC2) is an epigenetic regulator that trimethylates lysine 27 of histone 3 (H3K27me3) and is essential for embryonic development and cellular differentiation. H3K27me3 is associated with transcriptionally repressed chromatin and is established when PRC2 is allosterically activated upon methyl-lysine binding by the regulatory subunit EED. Automethylation of the catalytic subunit enhancer of zeste homolog 2 (EZH2) stimulates its activity by an unknown mechanism. Here, we show that human PRC2 forms a dimer on chromatin in which an inactive, automethylated PRC2 protomer is the allosteric activator of a second PRC2 that is poised to methylate H3 of a substrate nucleosome. Functional assays support our model of allosteric *trans*-autoactivation via EED, suggesting a previously unknown mechanism mediating context-dependent activation of PRC2. Our work showcases the molecular mechanism of auto-modification-coupled dimerization in the regulation of chromatin-modifying complexes.

## In brief

The multi-protein complex PRC2 is a transcriptional regulator that trimethylates lysine 27 of histone H3 (H3K27me3) upon EED-dependent allosteric activation. Here, using cryo-electron microscopy and functional assays, Sauer et al. show that PRC2 dimerization on chromatin enables stimulation via automethylated EZH2 binding to EED, enabling PRC2 activity independent of other activators.

## Graphical abstract

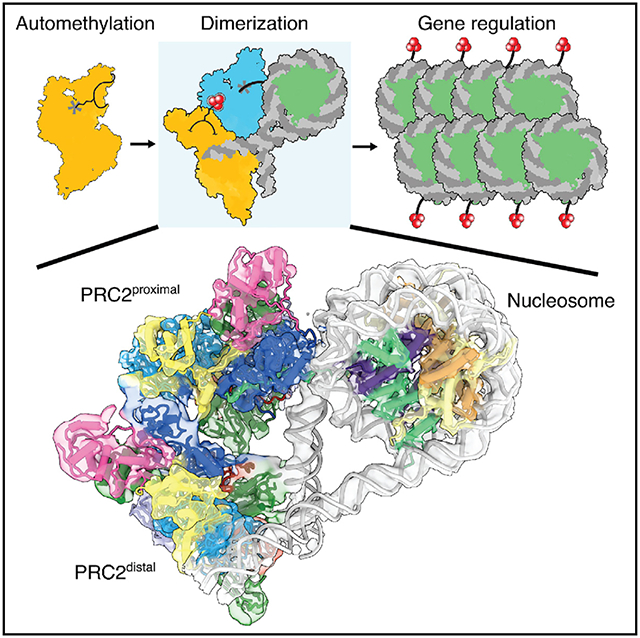

## INTRODUCTION

Polycomb repressive complex 2 (PRC2) is an essential chromatin regulator that harbors histone methyltransferase (HMTase) activity mediated by its catalytic subunit enhancer of zeste homolog 2 (EZH2). PRC2 is the only known factor that establishes the trimethylation of lysine 27 of histone H3 (H3K27me3), a chromatin mark associated with transcriptional repression and whose tight spatiotemporal regulation is essential during development and differentiation. In addition to EZH2 (or its less active homolog EZH1), the core PRC2 complex consists of suppressor of zeste 12 (SUZ12), retinoblastoma-associated protein 46 or 48 (RBAP46/48), and embryonic ectoderm development (EED).^1^ The binding of EED to trimethylated-lysine-bearing peptides is central to PRC2 activity since it allosterically activates EZH2, a requirement for efficient trimethylation of H3K27.^2^ Upon engagement of specific methylated peptides by EED, a flexible loop of EZH2, the stimulatory-response motif (SRM), folds into a short α-helix and stabilizes an active conformation of EZH2 by bridging the EED ligand binding site and the critical SET-I helix of the EZH2 catalytic su(var)3–9, enhancer-of-zeste, and trithorax (SET) domain.^3,4^ Two EED-binding ligands, themselves trimethylated by PRC2, have been described as functional activators: Jumonji/ARID domain-containing protein 2 (JARID2), an accessory PRC2 subunit that contributes to the targeting of the complex and *de novo* deposition of H3K27me3, and that binds EED through trimethylated JARID2 K116^5,6^; and H3K27me3 itself, which facilitates H3K27me3 propagation in a positive feedback loop.^2^ Highlighting the importance of this regulatory, allosteric mechanism, H3K27me3, and gene expression dynamics are impaired during development when EED-EZH2 communication is interrupted.^2,7,8^

Just like kinase activation can occur upon autophosphorylation,^9,10^ chromatin-modifying enzymes can also act upon themselves for regulation,^11,12^ sometimes in a manner that is linked to a change in oligomeric state (e.g., transcription factor-mediated dimerization and auto-acetylation of P300 have been suggested to be critical for target specificity and to act as molecular short-term memory during inflammatory response^13^). Recently, EZH2 has been shown to be automethylated in *cis*, leading to increased HMTase activity through a yet unknown mechanism.^14,15^ The three automethylated EZH2 residues, K510, K514, and K515, are part of a flexible loop (hereafter the automethylation loop, “am-loop”) that folds into an α-helix upon engagement with nucleosome substrates.^8,16^ This region is referred to as the “bridge helix” because it bridges the nucleosomal DNA, the substrate histone H3 tail, and the EZH2 SET domain.^8,16^

Here, we set out to investigate how automethylation of EZH2 may activate human PRC2 using single-particle cryo-electron microscopy (cryo-EM) and functional assays. To this end, we studied the structural implications of substrate chromatin engagement and automethylation in the absence of any allosteric activator other than automethylated EZH2, i.e., using mono-nucleosomes and recombinant PRC2 lacking the stimulatory subunit JARID2. We show that in a chromatin- and automethylation-dependent manner, PRC2 dimerizes such that an automethylated inactive PRC2 complex serves as an allosteric activator via the EED regulatory site of a second, substrate-engaged PRC2. Using separation of function mutants, we provide evidence that automethylation indeed regulates PRC2 activity in *trans* and that it functions in defined genomic contexts. Taken together, we propose that dimerization-dependent stimulation of PRC2 HMTase activity in *trans*, driven by EZH2 automethylation and local PRC2 concentration, represents a previously unknown mechanism to regulate the establishment of H3K27me3 heterochromatin domains.

## RESULTS

### Dimerization of automethylated PRC2 on chromatin

**Figure 1.**
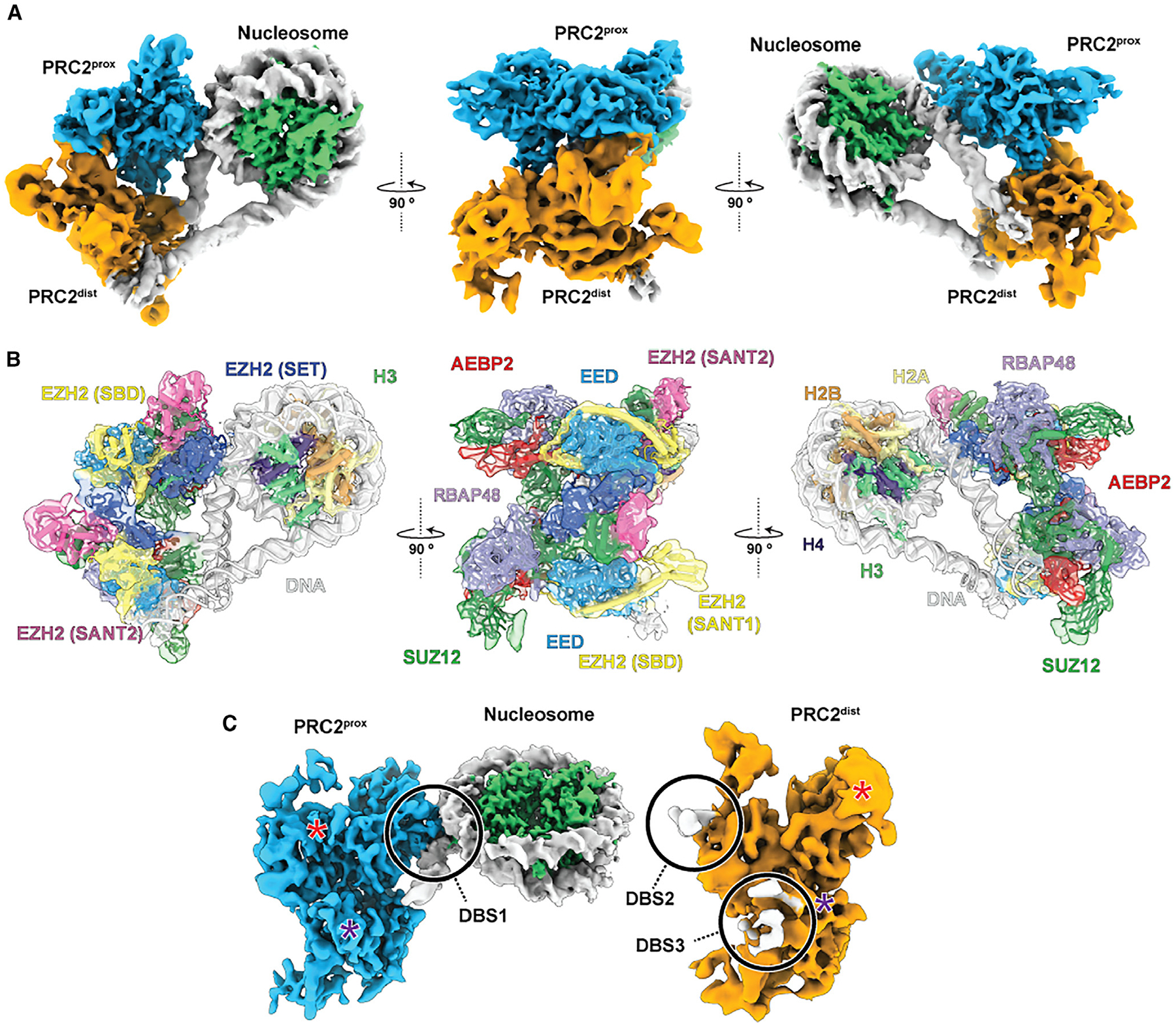
Characterization of an asymmetric PRC2 dimer bound to a nucleosome (A) Composite cryo-EM density for proximal (blue) and distal (orange) PRC2 complexes simultaneously engaged with a nucleosome (histones shown in green, DNA in gray) shown from three orthogonal orientations. (B) As (A), but with docked models colored by PRC2 subunit/domain (same colors used throughout). (C) The structure in (A) has been rotated and “opened” to show the proximal and distal PRC2 separated and in a “canonical” orientation. DNA-binding sites (DBSs) are indicated (see also Figure 2). Asterisks mark the dimer interfaces between EED^prox^ and EZH2^dist^ (red) and between SUZ12^prox^ and SUZ12^dist^ (purple).

To study the mechanism of EZH2 automethylation-mediated activation, we set out to solve the cryo-EM structure of PRC2 engaged with a substrate nucleosome in the absence of H3K27me3 or JARID2, and therefore with the am-loop as its only source of methylated peptides for possible allosteric activation. Mass spectrometry analysis of recombinantly expressed human PRC2, composed of EZH2, SUZ12, EED, RBAP48, and AEBP2, revealed EZH2 K510, K514, and K515 to be methylated to varying degrees. Incubation of recombinant PRC2 with S-adenosyl methionine (SAM) cofactor further increased the levels of am-loop automethylation (Figure S1). Cryo-EM analysis of automethylated PRC2 incubated with nucleosomes containing 40 bp stretches of linker DNA on both sides resolved two distinct species: nucleosomes bound by a single PRC2 complex (Figure S2) and two PRC2 complexes interacting with one nucleosome and with each other in a non-symmetrical dimer (Figures 1 and S3). Single nucleosome engaged PRC2 complexes were in a basal state in which PRC2 is not allosterically activated, as we previously described^16^ (i.e., folded bridge helix, but unfolded SRM; see later and Figure 4). Here, we will focus mainly on the observed PRC2 dimer-nucleosome complex (Figures 1A, 1B, S2–S5; Table S1).

The two PRC2 complexes and the nucleosome form a flexible, tripartite structure stabilized by contacts between the three components (Figure 1). In contrast to other recently described symmetric PRC2 dimers,^17,18^ the two PRC2 complexes are arranged asymmetrically, with only one of them contacting the histone octamer in a way that is compatible with H3 methylation. We refer to this PRC2 as nucleosome-proximal PRC2 (PRC2^prox^) (Figure 1A and 1C, blue). The second PRC2 is located distal from the histone core (referred to as PRC2^dist^) (Figures 1A and 1C, orange) and interacts distinctly with each of the two DNA linkers, as well as with the PRC2^prox^ (Figure 1). Employing 3D flexible refinement (3DFlex),^19^ we were able to characterize the extensive motions within this molecular arrangement (Figure S4). Despite the flexibility and transient nature of the complex, this strategy enabled us to unambiguously fit PRC2 and nucleosome models into an improved density map (Figure 1B), defining the inter-PRC2 and PRC2-DNA interfaces, as well as the distinct conformational states of the PRC2^prox^ and PRC2^dist^.

**Figure 2.**
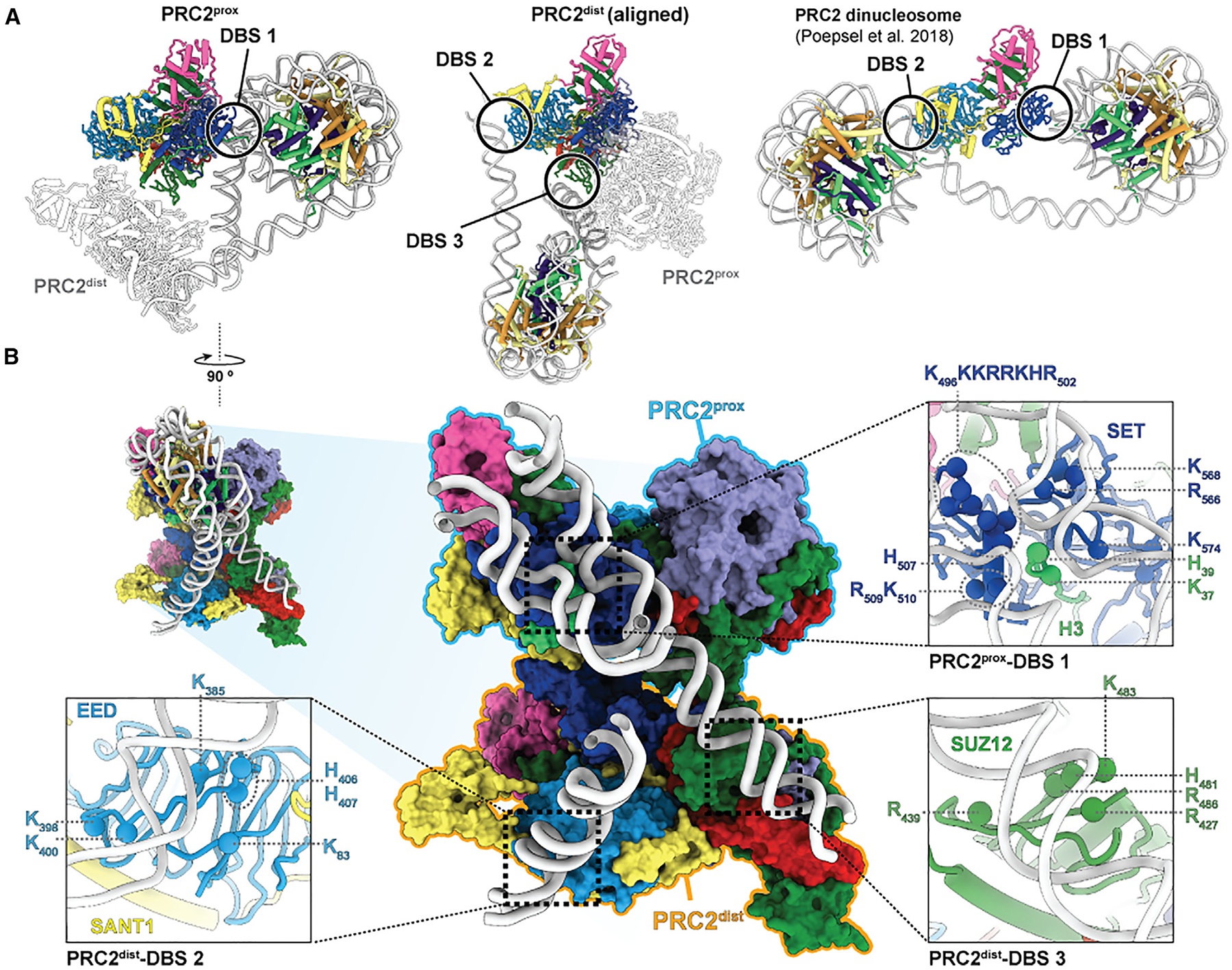
Three distinct DNA-binding sites are present in the PRC2 dimer-nucleosome structure (A) DNA interaction sites for PRC2^prox^ (left), PRC2^dist^ (center), and for PRC2 engaged with a dinucleosome (from Poepsel et al.^8^) (right) with all PRC2 complexes aligned to one another. The three distinct DNA-binding sites (DBSs) collectively observed are indicated as DBS1, DBS2, and DBS3. (B) PRC2 dimer viewed through the nucleosome (center; top left shows the relative orientation of the view shown with respect to the left panel in (A) before removing the histones and part of the DNA). The three different DBSs are displayed in more detail in the three zoom-out boxes. Positively charged residues within 10 Å of nucleic acid have been marked with spheres as possible sites of interaction with the phosphate backbone of the DNA.

In the PRC2^prox^, the SET domain of EZH2 engages the substrate H3 tail while its CXC domain contacts the nucleosomal DNA, as previously seen.^8,17,20^ We refer to this DNA-binding site (DBS), which involves the bridge helix containing the automethylated lysines, as DBS1 (Figures 1C; 2A, left; 2B). In the PRC2^dist^, a lateral surface of EED^dist^, which we refer to as DNA-binding site 2 (DBS2), binds one DNA linker (Figures 1C; 2A, center; 2B). The same site has been seen to bind DNA in the context of a hetero-dinucleosome substrate in which DBS2 binding of a H3K27me3-bearing nucleosome stimulates methylation of a substrate nucleosome engaged via DBS1 (Figure 2A, right).^8^ Thus, DBS2 in EED can engage either nucleosomal or linker DNA depending on the context. We additionally observe an unassigned cylindrical density contacted by a positively charged surface corresponding approximately to DBS2 in PRC2^prox^ (Figure S5) that may correspond to double-stranded DNA and is absent in the PRC2 monomer reconstruction (Figure S5B). PRC2^dist^ also contacts the other linker DNA, in this case via the neck region of SUZ12^dist^, a DBS of PRC2 that has not been described before and that we refer to as DBS3 (Figures 1C; 2A, center; 2B). Based on our docking into the cryo-EM density of PRC2^dist^, this DNA-binding interface likely involves the loop H_481_PKGA_485_ and several nearby positively charged residues of SUZ12 (Figure 2B). Therefore, PRC2 can utilize three distinct DNA-binding surfaces (DBS1–3) to mediate interactions with the local chromatin environment, and the three of them are involved in the tripartite engagement described in this study.

### Interaction between PRC2^prox^ and PRC2^dist^ and allosteric activation via the am-loop

**Figure 3.**
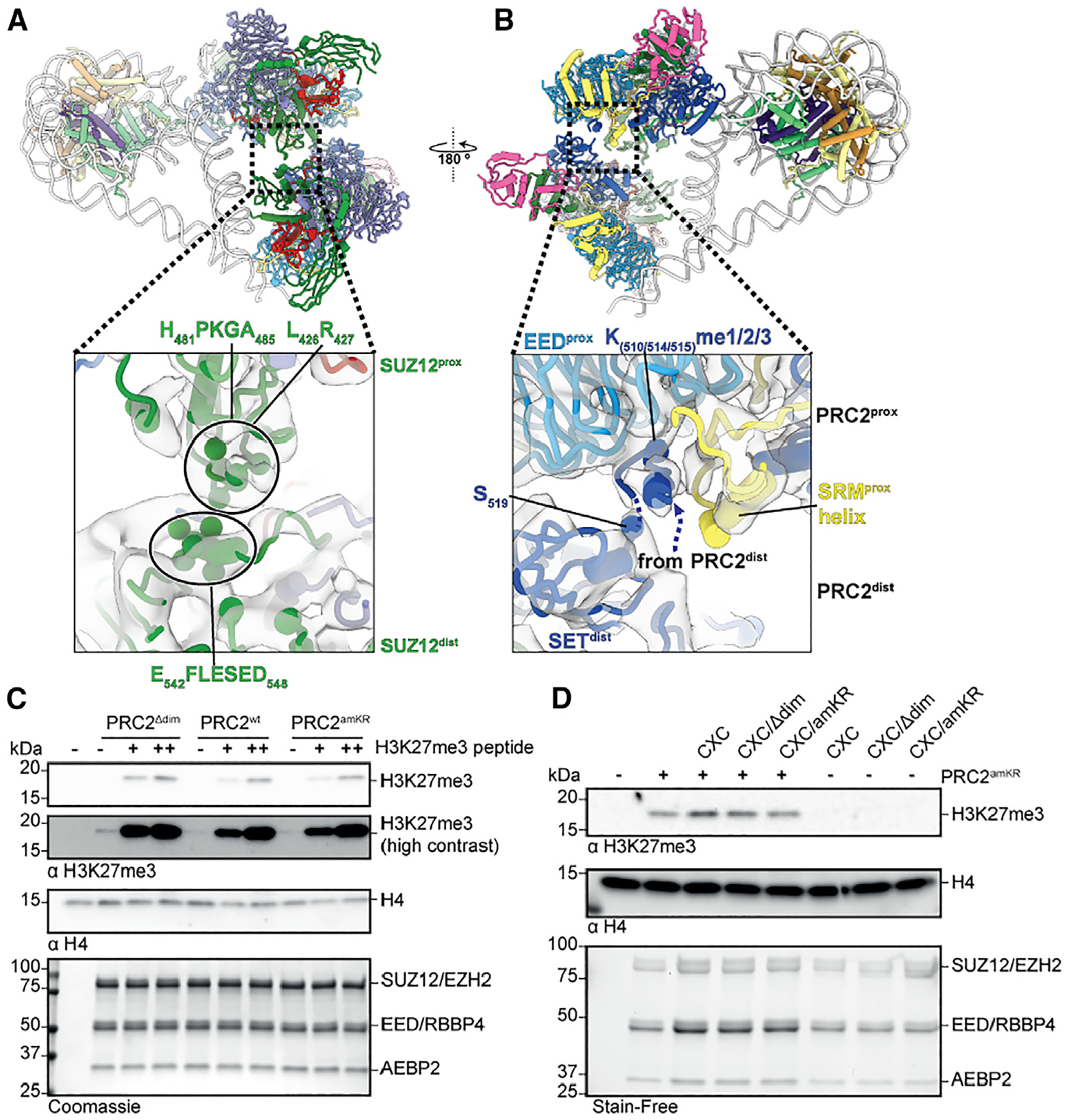
PRC2 dimer interfaces (A) Dimer interface involving SUZ12^prox^ and SUZ12^dist^. (B) Interaction of the SET domain of the PRC2^dist^ with the EED of PRC2^prox^. Potential interacting residues based on proximity are indicated by spheres in the zoom-out panels. The dashed lines in (B) indicate the likely direction of the disordered part of EZH2^dist^ connecting the methylated peptide with the rest of the SET^dist^. (C) HMTase assays on mononucleosomes. PRC2 variants were incubated with substrate nucleosomes either un-stimulated or in the presence of 1.5 μM (+) or 15 μM (++) H3K27me3 histone H3 peptide as allosteric stimulator. (D) Stimulation of PRC2 activity by PRC2^dist^. HMTase assays on mononucleosomes were carried out with PRC2^amKR^ in the presence of PRC2^CXC^ and dimerization- or automethylation-mutant variants (PRC2^CXC/Δdim^ or PRC2^CXC/amKR^).

In addition to their interactions with the nucleosome, the PRC2^dist^ and PRC2^prox^ interact with each other via two different interfaces. A SUZ12-SUZ12 interface comprises loop H_481_PKGA_485_ and a stretch that includes L426 and R427 of SUZ12^prox^. These elements engage with a short, negatively charged helix in SUZ12^dist^ (E_542_FLESED_548_) (Figure 3A). Thus, the region of SUZ12 involving loop H_481_PKGA_485_ and nearby residues appear to mediate two different interactions important within the supramolecular arrangement we are describing: one in PRC2^dist^ with linker DNA (DBS3), and one in PRC2^prox^ with another SUZ12 in PRC2^dist^.

**Figure 4.**
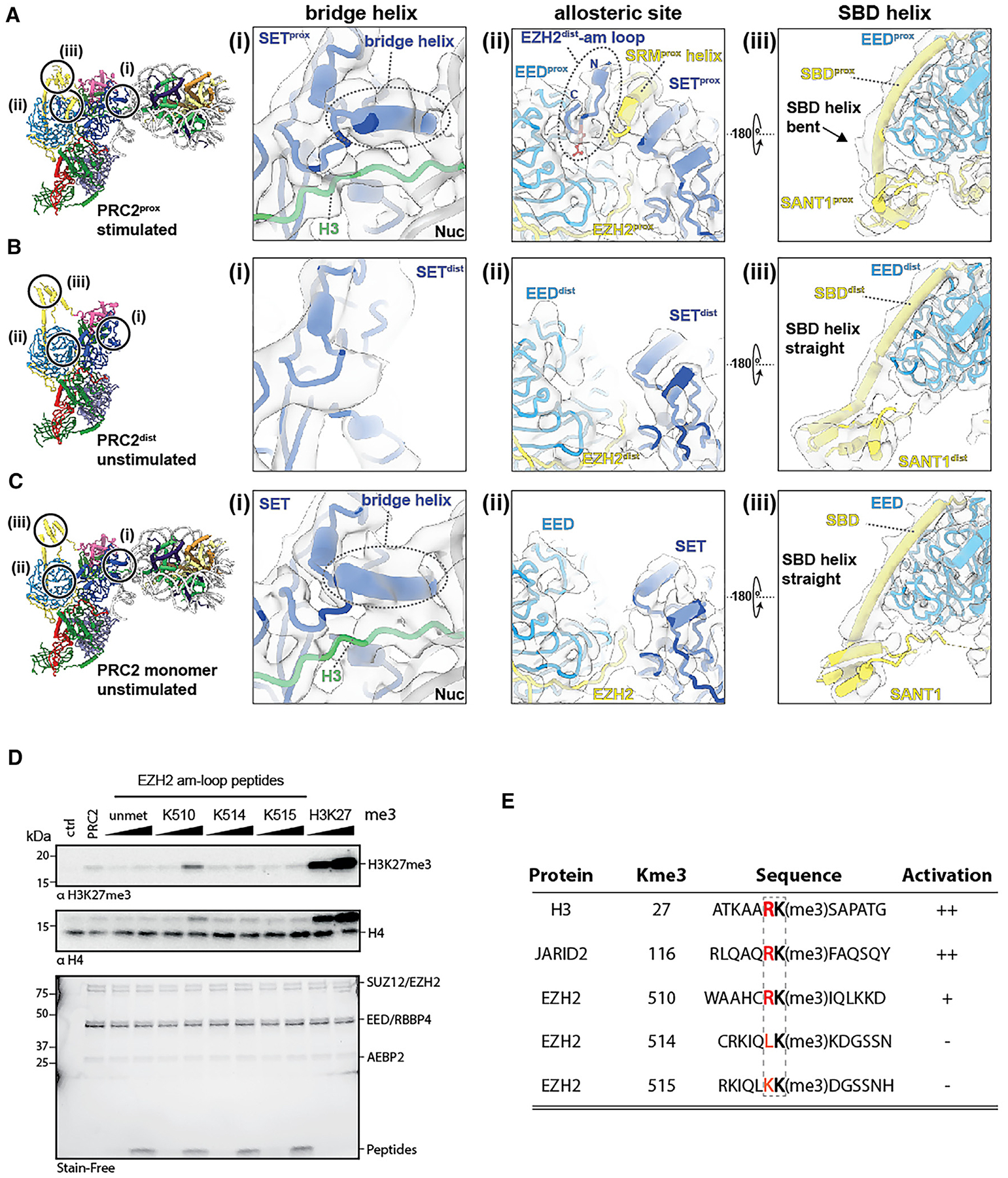
PRC2^dist^ allosterically activates PRC2^prox^ via its automethylated loop Close-ups showing: (i) the presence or absence of the bridge helix; (ii) the occupancy of the allosteric binding site on EED and the presence or absence of the SRM helix; and (iii) the conformation of the SBD helix for PRC2^prox^ (A), PRC2^dist^ (B), and PRC2 monomer (C). Only PRC2^prox^ is in an allosterically stimulated conformation, while PRC2^dist^ and PRC2 monomer are unstimulated. (D) HMTase assays on mononucleosomes in the presence of EZH2 am-loop peptides. PRC2^amKR^ was incubated with 200 nM substrate nucleosomes in the presence of 1.5 or 15 μM EZH2 peptide (aa502–521) either unmethylated (unmet) or containing trimethylated K510, K514, or K515. H3K27me3 peptide was used as a positive control. Ctrl corresponds to no PRC2. “PRC2” indicates no added peptide. (E) Comparison of the aa sequences surrounding the trimethylation site of EED-binding PRC2 activators and the EZH2 am-loop peptides. Residues at the −1 position relative to Kme3 are shown in red.

The second interaction between PRC2^dist^ and PRC2^prox^ involves the SET domain of EZH2^dist^ and EED^prox^ and has critical functional implications (Figures 3B and 4Aii). The EZH2^dist^ lacks density that would correspond to the bridge helix, indicating that the am-loop containing the automethylated K510, K514, and K515 is unfolded (Figure 4Bi). This observation was expected, given that PRC2^dist^ is not interacting with nucleosomal DNA, and is further supported by molecular dynamics (MD) analysis (see below). The SET domain of EZH2^dist^ is positioned close to EED^prox^ such that the automethylated lysines within the unfolded am-loop of EZH2^dist^ can easily reach the allosteric methyl-lysine binding pocket of EED^prox^ (Figure 3B). Indeed, local refinement shows clear density bound at the allosteric site of the EED^prox^ (Figures 3B and 4Aii). We conclude that this density corresponds to the am-loop of EZH2^dist^, which is the only source of methylated peptide in our sample. Accordingly, EZH2^prox^ shows an ordered SRM helix and a bent SANT binding domain (SBD) helix, two structural hallmarks of EED-mediated activation,^3,6^ showing that PRC2^prox^ is in an activated state (Figure 4Ai and 4Aiii). By contrast, neither EED^dist^ nor EED of the single PRC2-nucleosome structure reconstructed from the same dataset (which serves as an internal control) show density at the methyl-lysine binding site (Figures 4B and 4Cii). Accordingly, the SRM helix is absent from these reconstructions (and thus unfolded), and the SBD helix is in its straight conformation (Figures 4B and 4Ciii). Thus, while biochemically indistinguishable, PRC2^prox^ and PRC2^dist^ are in two distinct conformational and functional states: in PRC2^prox^, the bridge helix and SRM are both folded through their interactions with nucleosomal DNA and the occupied allosteric site of EED, respectively; in PRC2^dist^, those interactions are absent, and thus, both elements are unfolded (Figures 4A and 4Bi and 4Bii).

### Automethylation is required for *trans*-autoactivation of PRC2

Our structural data show that an automethylated PRC2 can serve as an allosteric activator in *trans* of a substrate-bound PRC2, inducing the hallmark structural features of allosterically activated EZH2. To investigate the functional implications of *trans*-activation, we generated a series of separation of function mutants. First, in PRC2^amKR^ we mutated the am-loop lysines to arginines to mimic a non-methylated am-loop (K510R, K514R, K515R) and saw that this mutant exhibits similar activity to the wild-type (WT) PRC2 complex, as previously described.^14^ Notice that, unlike an unmethylated WT loop, this mutant am-loop cannot bind the EZH2 active site as a substrate. In a second mutant, termed PRC2^Δdim^ hereafter, SUZ12 lacks the short helix E_542_FLESED_548_ involved in the SUZ12-SUZ12 dimer contacts (Figure 3A). Both PRC2 variants, PRC2^amKR^ and PRC2^Δdim^, did not display any defects in overall structure, nucleosome binding, or HMTase activity upon allosteric stimulation via H3K27me3 (Figures S6A–S6C), indicating that automethylation and dimerization are not required for PRC2 activity when it is stimulated by an excess of an alternative allosteric activator (Figure 3C). *In vitro* HMTase activity assays to analyze allosteric activation mediated by PRC2 dimerization are complicated by the fact that PRC2 generates its own allosteric activator, i.e., H3K27me3. To address this, we made use of a third separation of function mutant that carries mutations in the CXC domain of EZH2 (K568A/Q570A/K574A/Q575A, “PRC2^CXC^”) that disrupt nucleosome engagement and abrogate HMTase activity on nucleosomes.^8,21^ Recombinant PRC2^CXC^ was automethylated as confirmed by mass spectrometry (Figure S6D) and should still be able to adopt the distal position in a dimer to serve as a *trans*-activator for a PRC2^prox^. Simultaneously, we used the PRC2^amKR^ mutant as the nucleosome-modifying enzyme, which cannot itself serve as an allosteric activator. In agreement with our model, PRC2^CXC^ alone exhibited undetectable activity but increased the amount of H3K27me3 generated by PRC2^amKR^ by ~50% (Figures 3D and S6E). Thus, although PRC2^CXC^ cannot methylate nucleosomes, it can stimulate HMTase-competent PRC2. Disrupting automethylation or dimerization by combining the CXC with either am-loop (PRC2^CXC/amKR^) or SUZ12 dimer interface mutations (PRC2^CXC/Δdim^), respectively, abrogated HMTase stimulation (Figures 3D and S6D). Together with our structural data, these functional assays suggest that the methylated am-loop activates PRC2 within a dimer via the EED trimethyl-lysine binding site.

### *Trans*-activation is mediated by EED

To further specify the significance of EED binding for this activation mechanism, we used either recombinant PRC2^amKR^ additionally harboring a “cage mutant” EED Y365A subunit deficient in methyl-lysine binding (PRC2^amKR/EEDc^)^2^ or an EED inhibitor (MAK683)^22^ as two independent approaches to disrupt allosteric activation via EED. In both assays using the EED-mutant or in the presence of the small molecule inhibitor, H3K27me3 was strongly reduced, irrespective of the addition of PRC2^CXC^ (Figure S7A). These results are in agreement with our model that stimulation by dimerization is mediated by EED.

In contrast to what we observed for H3K27me3, H3K27 dimethylation (H3K27me2) was not increased upon addition of PRC2^CXC^ (Figure S7B). It is well established that basal H3K27 mono- and dimethylation by PRC2 is efficient even in the absence of allosteric activators, which are only required for the rate-limiting conversion of H3K27me2 to me3.^23,24^ Therefore, our results confirm that the stimulatory effect of automethylated PRC2^CXC^ by dimerization affects the rate-limiting, EED-dependent step of H3K27 trimethylation.

To investigate the possible relative contribution of each of the methylated lysines in the EZH2 am-loop to the allosteric activation, we performed further *in vitro* HMTase assays against recombinant nucleosomes using synthetic am-loop peptides as possible activators. Interestingly, a stimulatory effect was only detectable for EZH K510me3, whereas either K514me3 or K515me3 on their own or unmethylated peptides did not affect HMTase activity under the conditions used (Figure 4D). Analysis of sequence alignment for known allosteric activators of PRC2 via EED suggests that an Arg residue at the −1 position relative to the methyl-lysine is required for their activity, likely to stabilize the allosteric SRM helix.^2,4^ Such analysis agrees with our results, since of all of the automethylated am-loop lysines, only K510 has an Arg at the −1 position (Figure 4E). Taken together, our results show that EZH2 automethylation via dimerization results in allosteric *trans*-autoactivation of PRC2 via EED to enable H3K27 trimethylation, and that EZH2 K510me3 is the primary am-loop modification that exerts this effect.

### Impact of automethylation on bridge helix folding and nucleosome engagement

**Figure 5.**
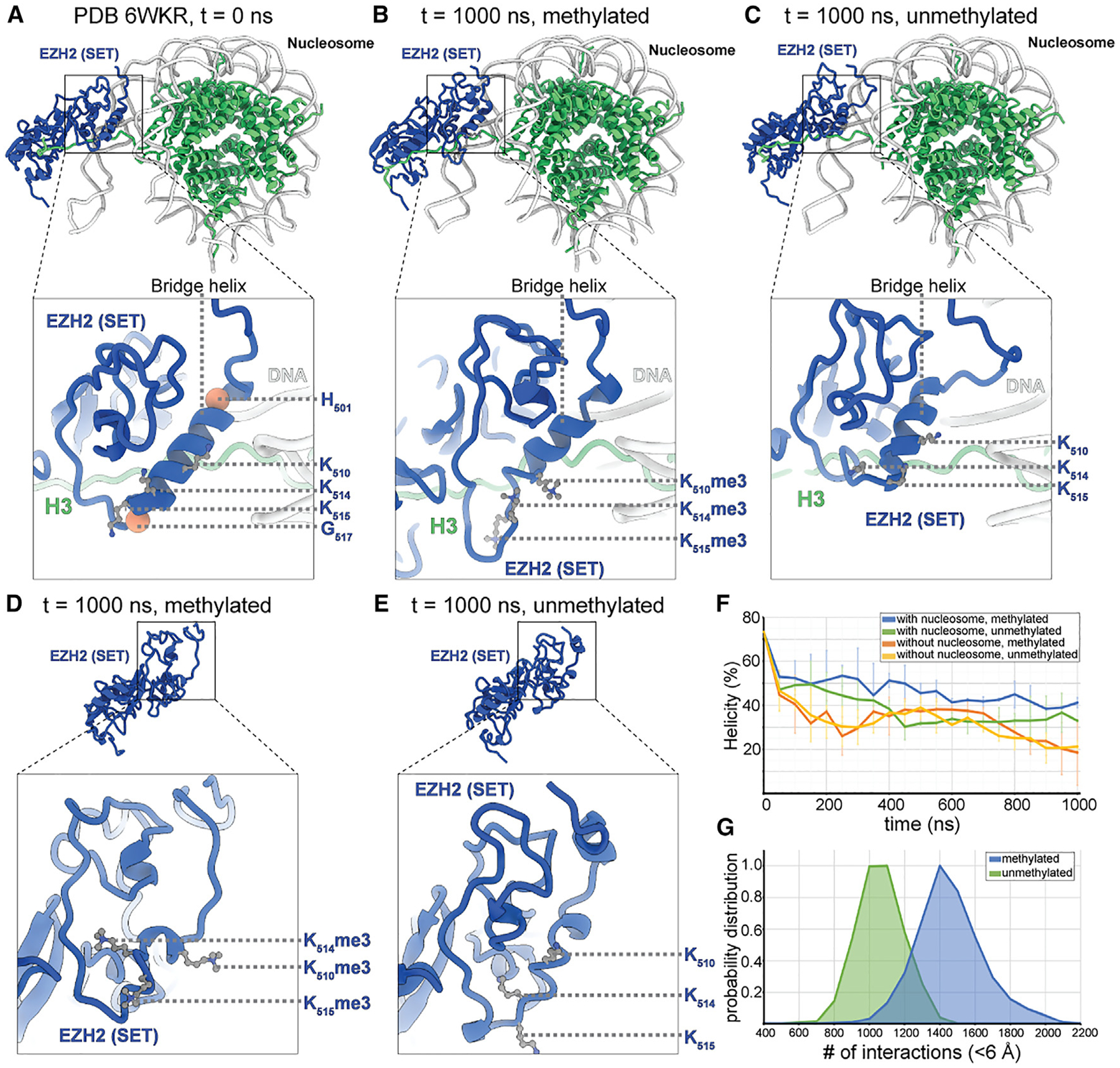
MD simulations of the EZH2 SET domain in complex with the nucleosome, analyzing the impact of auto-methylation on the conformational dynamics of the bridge helix (A–C) Representative simulation snapshots of EZH2 SET domain bound to a substrate nucleosome at t = 0 (A) and at t = 1,000 ns with the am-loop lysines K510, K514, and K515 trimethylated (B) or unmethylated (C). t = 0 corresponds to PDB: 6KWR. Blue, EZH2 SET domain; green, histone proteins. The three automethylated lysines are shown in ball and stick representations. H501 and G517 are shown as coral spheres. (D and E) Snapshot of EZH2 SET domain simulation performed in the absence of nucleosome at t = 1,000 ns with the am-loop trimethylated (D) or unmethylated (E). Each simulation has been performed in two replicates. (F) Analysis of the helicity of the bridge helix, including residues H501-G517 over a simulation period of 1,000 ns. Helicity has been calculated as average from two technical replicates with error bars showing standard deviation. (G) Analysis of low-distance (<6 Å) contacts between the H3 tail and the SET domain accumulated over the course of the 1,000 ns simulation for methylated (blue) or unmethylated (green) bridge helix. Data are averaged from two experiments each.

In addition to the *trans*-regulatory effect exerted by means of PRC2 dimerization, automethylation could act in *cis* by affecting the conformational dynamics of the bridge helix, with potential effects on substrate nucleosome binding and histone tail engagement. To analyze a possible relationship between automethylation and bridge helix folding, we performed MD simulations for two distinct scenarios: (1) no methylation and (2) tri-methylation of K510, K514, and K515, both in the presence and absence of nucleosome. Consistent with the disorder-to-order transition of the bridge helix indicated by cryo-EM experiments,^8,16^ our simulations show that the helix is more stable when the EZH2 SET domain is nucleosome-bound (Figures 5A–5C) as compared with the unbound scenario (Figures 5D and 5E). All simulations show an initial decrease in helicity for the bridge helix, but an overall higher degree of helicity is maintained for nucleosome-bound cases (Figure 5F). Variability between technical replicates, however, indicates dynamic conformational behavior of the bridge helix in all setups that is consistent with minimal influence of methylation on the stability of the bridge helix (Figure 5F). On the other hand, simulation data suggest that automethylation may increase the overall probability of the SET domain to interact with the H3 tail, potentially facilitating substrate engagement (Figure 5G). Overall, our MD simulations support bridge helix stabilization upon nucleosome binding but do not show a significant effect of automethylation on bridge helix folding and only a small effect on H3 tail engagement. Thus, automethylation likely does not have a major impact on PRC2 function in *cis* by altering the dynamics of the bridge helix.

### Role of automethylation in PRC2 gene silencing function

**Figure 6.**
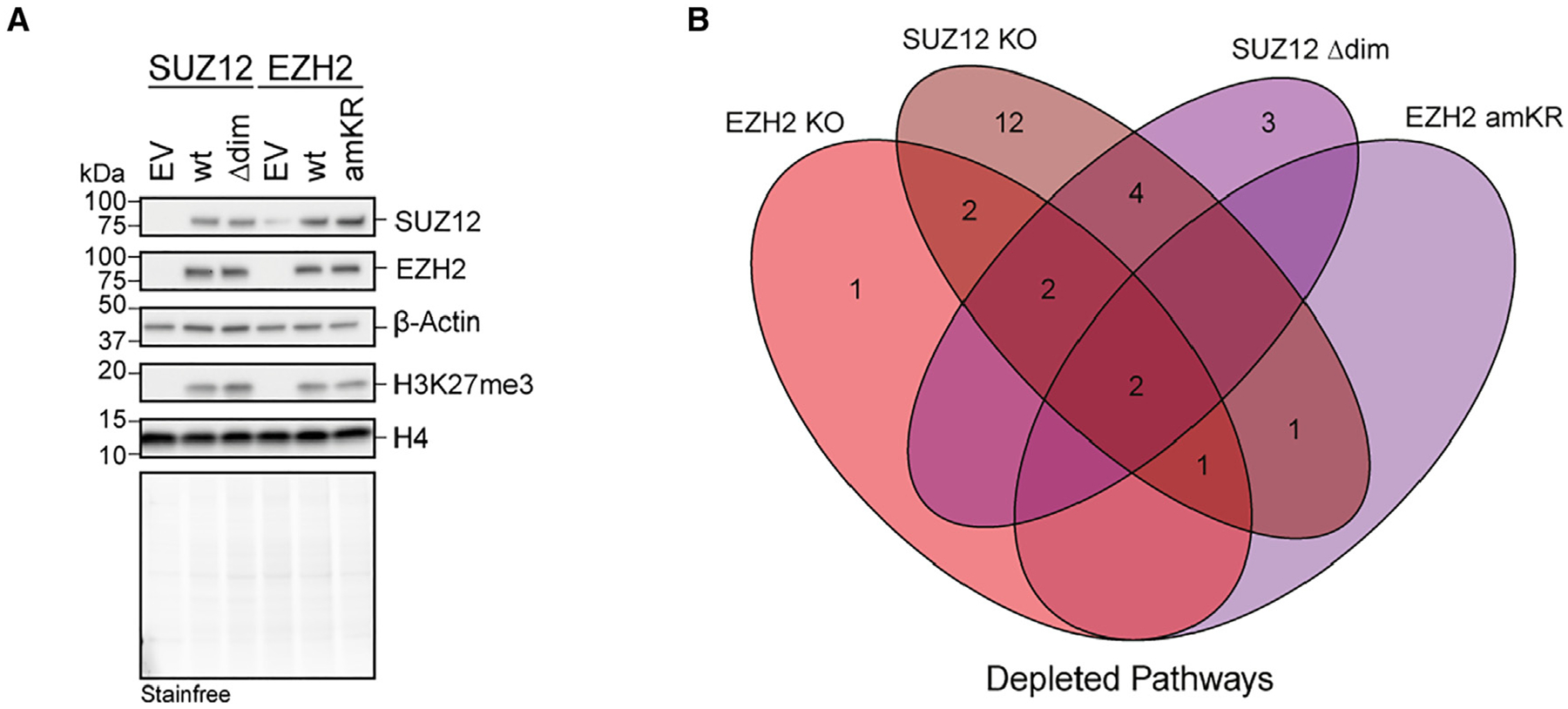
Impact of PRC2 automethylation on transcriptional dynamics during RA-induced differentiation of mESCs (A) Western blot using the indicated antibodies of whole-cell lysates obtained from SUZ12 KO mESCs stably transfected with an empty vector (EV) or expressing SUZ12 WT or SUZ12^Δdim^, and from EZH1/2 dKO mESCs stably transfected with an empty vector (EV) or expressing EZH2 WT or EZH2^amKR^. (B) Venn diagram illustrating the overlap of transcriptional changes according to gene set enrichment analyses (GSEAs) of RNA-seq data at day 4 of all-*trans* retinoic acid (ATRA) induced differentiation. Depleted pathways in EZH2dKO or SUZ12 KO, as well as the EZH2^amKR^ and SUZ12^Δdim^ rescue cells relative to the respective WT rescue are shown.

Based on our cryo-EM and biochemistry results, disruption of automethylation and dimerization could impact PRC2 function in transcriptional regulation by interfering with *trans*-activation. In particular, our studies would suggest that automethylation could have an effect on the initiation of PRC2 activity in regions in which *trans*-activation will be the only mode of EED-based activation in the absence of other allosteric modulators of PRC2 (i.e., JARID2 or methylated nucleosomes nearby). We therefore aimed to test the impact of automethylation and dimerization by rescue experiments in murine embryonic stem cells (mESCs). In EZH1/2 double knock-out (dKO) mESCs,^25^ the expression of either WT EZH2 or automethyl-mutant EZH2^amKR^ was stably restored, and in parallel, WT SUZ12 or SUZ12^Δdim^ were expressed in SUZ12 knock-out (KO) mESCs.^26^ Restored expression of either WT or mutant EZH2 or SUZ12 in this system led to comparable bulk levels of H3K27me3 (Figure 6A). This observation is consistent with previous results showing that upon sustained expression at endogenous levels, WT and automethyl-mutant PRC2 achieve similar H3K27me3 levels in bulk.^15^ For first insights into the extent by which automethylation and dimerization affect PRC2 function, we imposed a differentiation stimulus by all-*trans* retinoic acid (ATRA) on KO and rescue mESCs and compared their transcriptomes by RNA sequencing (RNA-seq) as a read-out of PRC2 function in transcription regulation. All cell lines were capable of lineage commitment without evidence of a substantial delay in differentiation as judged by pluripotency and neuronal lineage marker gene expression (Figures S8A and S9A). However, particularly at day 4 of ATRA treatment, a later time in differentiation, we noticed several differentially expressed genes (DEGs) in the rescued mutants (EZH2^amKR^, SUZ12^Δdim^) and KO cell lines as compared with WT mESCs (Figures S8C and S9C). The larger number of DEGs in KO versus mutant rescue cell lines suggests that PRC2^amKR^ and SUZ12^Δdim^ are, at least partially, capable of restoring PRC2 function but fail to rescue the expression level of a number of transcribed regions relative to the WT state. To reveal a potential defect caused by the mutant PRC2 subunits, we performed gene set enrichment analyses (GSEAs) to uncover pathways that are differentially regulated between the EZH1/2 dKO or EZH2^amKR^ relative to WT EZH2, as well as between SUZ12 KO or SUZ12^Δdim^ relative to WT SUZ12 rescue cells. The EZH1/2 dKO and EZH2^amKR^ cells share 21 out of 29 (EZH1/2 dKO) or 28 (EZH2^amKR^) enriched pathways, and three out of 8 (EZH1/2 dKO) or 4 (EZH2^amKR^) depleted pathways, suggesting that the EZH2^amKR^ mutant functionally overlaps with the loss of PRC2 in some contexts. Pathways that were enriched include, for example, the inflammatory response and the stress response, while pathways for cell division and checkpoint progression were depleted (Figures 6B and S8C). In addition, the SUZ12^Δdim^ rescue showed depletion of 11 pathways compared with the SUZ12 WT rescue cells, out of which 8 were also depleted in the SUZ12 KO cells. Affected pathways shared between the SUZ12 KO and SUZ12^Δdim^ included the stress response and pre-oncogenic pathways (Figures 6B and S9C). Together, these analyses suggest that mutations that interfere with automethylation or dimerization affect the transcriptional outcome of PRC2 function and that the affected pathways partially overlap between these mutants and the loss of PRC2. Of note, two depleted pathways relative to the WT rescue—E2F targets and G2M checkpoint—were shared by the PRC2 mutants and both KO states. These de-regulated pathways are in agreement with a described dependency of retinoblastoma protein (RB) signaling on polycomb function.^27^ The shared functional consequence of either disrupting automethylation or dimerization further supports a dimerization-dependent activation of automethylated PRC2. Together, our findings suggest a context-specific role of automethylation and dimerization, likely one in which activation cannot occur via pre-existing H3K27me3 or methylated JARID2, and PRC2 activity is dependent on *trans*-autoactivation.

### Chromatin context of *trans*-autoactivation

Our genomic analysis led us to propose that different contexts will involve alternative modes of PRC2 activation via EED. It is known that the subunit composition of variant PRC2 complexes affects the targeting and function of PRC2 and constitutes an important layer of its regulation. For example, JARID2 recruitment to chromatin via the engagement of mono-ubiquitinated histone H2A (H2A K119ub)^16^ or long non-coding RNAs^28^ is a possible mechanism to specify *de novo* H3K27 trimethylation in the genome. Interestingly, we were not able to detect nucleosome-bound PRC2 dimers when JARID2 was present in the complex, even when using a JARID2 construct that lacked its lysine 116 methylation site (PRC2_J119–450_) that would otherwise compete with PRC2^dist^ binding to EED. Comparison of different PRC2 structures shows that even in the absence of K116me3, JARID2 likely outcompetes PRC2^dist^ by sterically blocking the SUZ12-SUZ12 dimerization interface and/or impeding linker DNA binding by PRC2^dist^ (Figure S10A). Similarly, dinucleosome engagement of PRC2 representing an H3K27me3 spreading site, in which an H3K27me3-bearing nucleosome allosterically activates EZH2 to methylate an adjacent nucleosome via EED,^8^ is sterically incompatible with dimerization as shown here. In a hetero-dinucleosome (one allosterically activating, one substrate of PRC2), the methylated nucleosome would clash with the SANT2 domain of the PRC2^dist^ (Figure S10B). This analysis further supports the idea that allosteric *trans*-activation by dimerization is a context-specific mechanism of PRC2 activation that occurs as an alternative, not in addition, to activation by JARID2 K116me3- or H3K27me3-bearing nucleosomes.

The linker histone H1 is another determinant of chromatin context that has been proposed to cooperate with PRC2 to suppress gene expression through an unknown mechanism.^29^ Interestingly, cryo-EM analyses of PRC2 incubated with H1-bearing nucleosomes suggest that H1 binding and PRC2 dimerization are mutually exclusive since all reconstructions of PRC2 dimers lacked density for H1 (Figure S11A). On the other hand, we could see clear EM density corresponding to H1 when a PRC2 variant containing JARID2_119–450_ was used, a condition that prevents PRC2 dimerization (Figures S11B and S11C). Notice that this PRC2_J119–450_ is seen in an inactive conformation, as expected given the absence of an allosterically activating methylated peptide, and also that there is no direct interaction between H1 and PRC2_J119–450_. Comparison of the nucleosome-H1, nucleosome-H1-PRC2_J119–450_, and nucleosome-PRC2^prox^-PRC2^dist^ structures shows that H1 binding gives rise to a linker DNA trajectory that is not compatible with PRC2^dist^ binding (Figures S11D and S11E). Therefore, these observations indicate that genomic occupancy of H1 is incompatible with the *trans*-autoactivation of PRC2 by dimerization and agree with the latter being context specific as indicated by our genomic data.

## DISCUSSION

### An extended model for *de novo* establishment of H3K27me3

**Figure 7.**
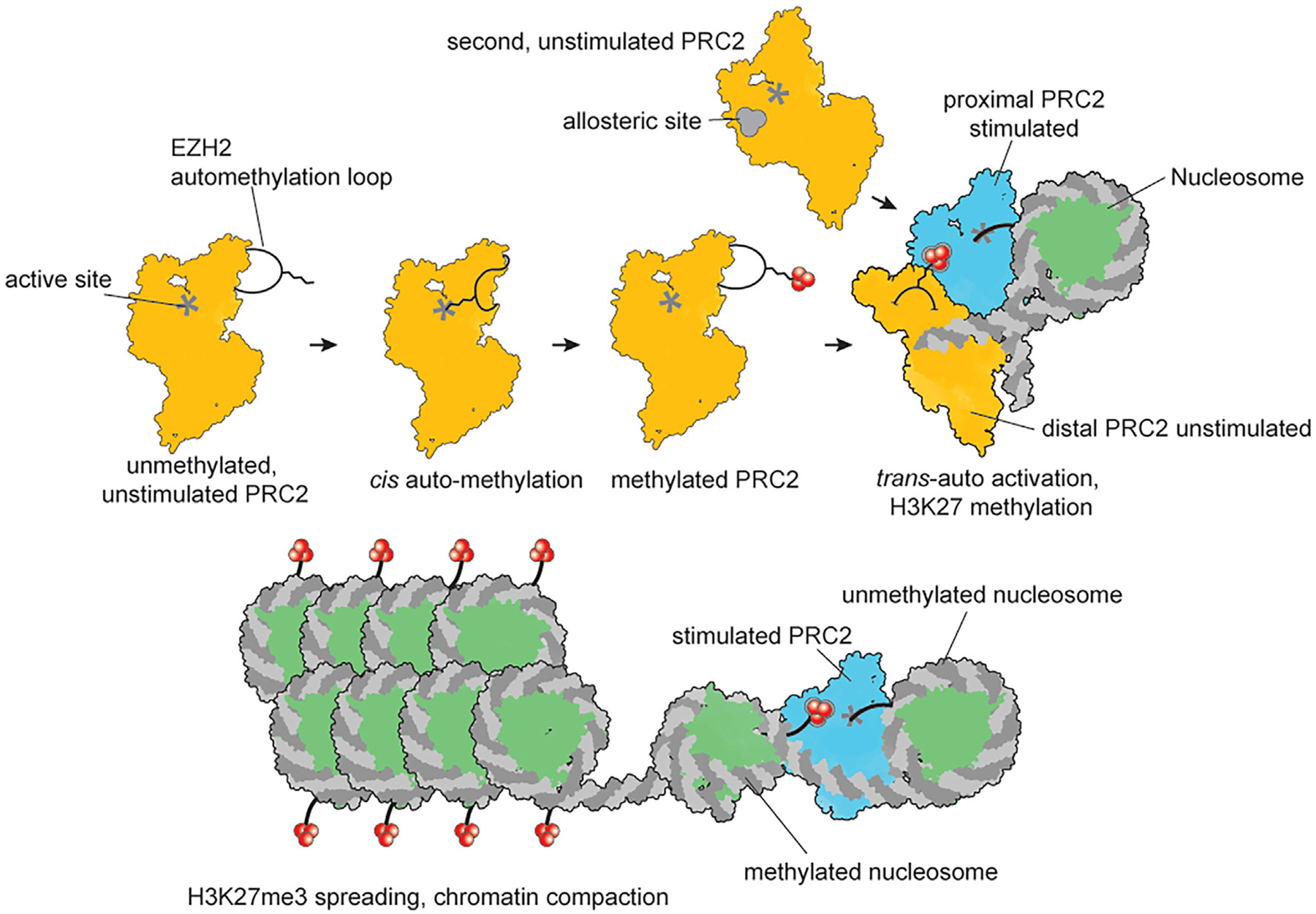
*Trans*-autoactivation model PRC2 that undergoes automethylation in *cis* can act as an allosteric activator in *trans* for a second PRC2 that then methylates H3 on the nucleosome as they interact with each other in the context of chromatin. Once nucleosomes containing H3K27me3 accumulate, PRC2 can be allosterically activated by methylated nucleosomes to further spread the H3K27me3 mark and ultimately cause chromatin compaction. We propose that *trans*-autoactivation of PRC2 enables initiation of H3K27me3 domains in the absence of other stimulating cofactors.

The activity of the PRC2 chromatin regulator is critical in development to both establish and maintain cell identity. Key to its function are the regulation of its genomic targeting and the local regulation of its HMTase activity. The discovery of the stimulatory effect of EZH2 automethylation on HMTase activity^14,15^ led us to hypothesize that automethylated EZH2 may act via the well-established allosteric methyl-lysine binding site in the regulatory subunit EED. To investigate the underlying mechanism, we studied the impact of this modification on PRC2 conformation and nucleosome engagement in the absence of any other methylated peptide. Our studies led us to discover a chromatin-dependent PRC2 dimer in which the automethylated am-loop of a PRC2^dist^ binds the allosteric site in EED of a PRC2^prox^ that is engaged with the tail of the substrate nucleosome (Figure 7). The two PRC2 complexes are present in two distinct conformational states, with only PRC2^prox^ showing the structural hallmarks of allosteric activation (a bent SBD helix and stabilized SRM). In agreement with the model of PRC2 regulation that emerges from these structural observations, functional assays using separation of function mutants show that nucleosome-binding deficient PRC2 (PRC2^CXC^) can serve as an allosteric activator, as long as automethylation and dimerization are not impaired. Furthermore, we could confirm that am-loop methylation mediates allosteric activation via EED and that this activation is caused by EZH2 K510me3, one out of three possibly methylated lysine residues of the am-loop.

The *trans*-activation mechanism demonstrated here employs the established EED-EZH2 allosteric communication axis that has been well studied for other activators, i.e., H3K27me3 and JARID2 K116me3. In all three cases, the methylated peptide binds EED and causes the stabilization of the SRM and the active conformation of the EZH2 SET domain, underscoring the central role of this mechanism in PRC2 regulation. Therefore, it is expected that the activation by all three established EED ligands represents alternative pathways of PRC2 activation. In agreement with this notion, the *in vitro* HMTase activity of automethylation or dimerization mutant PRC2 is unaffected when stimulated by an excess of H3K27me3 peptide. Additionally, we show that *trans*-autoactivation by dimerization and activation by JARID2 K116me3 or H3K27me3 are mutually exclusive, since H3K27me3-bearing nucleosomes would clash with PRC2^dist^, and no PRC2 dimers were observed when JARID2 containing PRC2 variants were used. We conclude that dimerization-mediated autoactivation is an EED-dependent mechanism of PRC2 activation that occurs at genomic loci in which automethylated EZH2 is the only available activator of PRC2. This model is supported by our transcriptomics analyses in mESCs showing that the abrogation of automethylation or interfering with dimerization affects transcriptional programs, e.g., E2F targets and the G2M checkpoint, at a later stage of our differentiation model, showing a functional overlap of de-regulated pathways under these conditions. We conclude that automethylation-mutant EZH2 fails to establish PRC2 function in distinct genomic contexts rather than globally. This context dependence is incompatible with a strong *cis*-regulatory effect of EZH2 automethylation, which would affect PRC2 activity independent of context. In agreement with the absence of a strong *cis*-regulatory mechanism of automethylation that could affect bridge helix folding and/or substrate nucleosome engagement, our MD analyses show minimal effects of automethylation on bridge helix folding and only a limited impact on histone tail engagement by PRC2. Accordingly, nucleosome binding was not strongly affected by am-loop mutations in PRC2^amKR^, and previous work showed that automethylation-mutant PRC2 does not show a defect in chromatin engagement and genome-wide occupancy.^14^

It has been proposed that the unmethylated am-loop could bind the active site of EZH2 competing with other substrates, and thus, am-loop methylation could release an auto-inhibited state of PRC2.^15^ In the context of this *cis* hypothesis, the PRC2^amKR^ mutant simulates a state in which the auto-inhibition is released, thus resembling the methylated am-loop. Under this assumption, one would expect increased activity of PRC2^amKR^, which is not observed. Such a hypothesis also cannot explain the *in vitro* stimulation by PRC2^CXC^ of PRC2^amKR^. While we cannot exclude a possible impact of am-loop binding to the EZH2 active site on PRC2 activity, our work indicates that mechanisms acting in *cis* alone are insufficient to explain the stimulatory effect of EZH2 automethylation on PRC2 activity.

In a physiological context, the *de novo* establishment of H3K27me3 can be triggered via PRC2 activation by methylation of JARID2 K116, which is recruited to genomic loci via H2A K119ub^16,30^ or long non-coding RNAs.^5,28^ We propose that PRC2 dimerization could initiate H3K27me3 in the absence of JARID2, e.g., in cells that lack JARID2 expression or at genomic loci that do not recruit JARID2. Unlike activation by JARID2, dimerization requires two PRC2 complexes, thus higher local PRC2 concentration. This likely implicates local concentration levels of PRC2 in regulating initial H3K27me3 deposition, e.g., involving factors that recruit but not themselves activate PRC2. Further context specificity is suggested by our observation that histone H1 binding and *trans*-autoactivation by dimerization appear incompatible. These findings underscore the intricate ways in which chromatin regulators integrate cues from the local chromatin environment for their targeted and regulated function. Our work showcases the central role that the regulatory EED subunit has in instructing PRC2 HMTase activity in diverse contexts. Moreover, *trans*-autoactivation by dimerization of PRC2 shows that mechanisms that integrate auto-catalysis, homooligomerization, and allosteric regulation are not limited to the classical examples of kinase autophosphorylation^9,10^ but are highly relevant to multi-protein complexes that regulate chromatin function via histone modification.

Homotypic interactions of enzymes that are regulated through auto-catalysis can have various functional implications, including the amplification of regulatory signals, impact on substrate recognition, and additional layers of regulation, such as feedback loops or temporal control. EZH2 automethylation likely enables the *de novo* deposition of H3K27me3 in distinct chromatin contexts. One could even imagine that the stable methylation of lysines in the am-loop of EZH2 may act as a molecular memory of PRC2 activity, potentially enabling *trans*-activation of multiple complexes. Furthermore, the system may be further fine-tuned by combining different variants of PRC2 within one dimer. For example, EZH1-containing complexes, which show little HMTase activity themselves, could potentially serve as allosteric activators in hetero-dimers of PRC2/EZH1 and PRC2/EZH2, since the am-loop is conserved between the two proteins. The overall ability of additional cofactors and subunit variants to affect PRC2 targeting and to facilitate or impede the formation of PRC2 dimers remains to be investigated. Another open question is whether and how automethylation, a prerequisite for dimerization-mediated activation, is itself regulated. Thus, automethylation and allosteric dimerization add further layers of complexity to PRC2 targeting and regulation and provide support for the critical role played by trimethyl-lysine binding to the regulatory subunit EED. Future work will determine whether regulation by dimerization extends to other key epigenetic factors that have been shown to auto-modify.^11–13^

### Limitations of the study

Our structural and biochemical analyses strongly support a model in which automethylation mediates allosteric activation in *trans* in the context of chromatin-bound PRC2 dimers. However, several open questions still need to be addressed in future functional studies, such as which are the biological contexts in which dimerization-mediated auto-activation is required for PRC2 function, what are the genomic regions affected by this form of PRC2 regulation, and what are its ultimate consequences on cellular differentiation and other PRC2 functions.

## RESOURCE AVAILABILITY

### Lead contact

Further information and requests for reagents should be directed to and will be fulfilled by the lead contact, Eva Nogales (enogales@lbl.gov).

### Materials availability

Plasmids and strains generated in this study are available upon request from the lead contact with a completed Materials Transfer Agreement.

### Data and code availability

- Cryo-EM raw data have been deposited at EMPIAR, cryo-EM density maps at Electron Microscopy Data Bank (EMDB), atomic models at the Protein Data Bank (PDB), RNA-seq data at NCBI’s Gene Expression Omnibus (GEO), and proteomics data for the analysis of PRC2^CXC^ and PRC2^CXC/Δdim^ at the ProteomeXchange Consortium via the PRIDE^31^ partner repository (https://www.ebi.ac.uk/pride/archive/). Raw gel and immunoblotting images are available at Mendeley Data, doi: https://doi.org/10.17632/m65ctymnbf.1. All these data are publicly available as of the date of publication. Accession numbers are listed in the key resources table.
- This paper does not report original code.
- Any additional information required to reanalyze the data reported in this paper is available from the lead contact upon request.

## STAR★METHODS

### EXPERIMENTAL MODEL AND STUDY PARTICIPANT DETAILS

#### Insect cell strains

Baculoviral stocks were generated in Sf9 cells (Expression Systems) cultivated at a starting density of 0.5 × 10^6^ cells/ml in uncoated tissue culture dishes at 27 °C using protein free ESF 921 insect cell culture medium. For protein expression, *Trichoplusia ni* cells (Tni, Expression Systems) were infected with baculovirus at a density of 1 × 10^6^ cells at 27 °C in a shaker incubator, expression was done for 72 hours prior to harvest.

#### Murine embryonic stem cell lines

MESCs were cultivated on 0.1% gelatin-coated dishes inGMEM supplemented with 20% ES-grade fetal bovine serum 2 mM glutamine, 100 U/ml penicillin, 0.1 mg/ml streptomycin, 0.1 mM non-essential amino acids, 1 mM sodium pyruvate, 50 μM ß-mercaptoethanol, 1000 U/ml ESGRO Leukemia Inhibitory Factor, 3 μM GSK3β, 1 μM MEK 1/2 inhibitors at 37°C, 95% humidity and 5% CO2. The EZH1/2 dKO mESCs (E14TG2a) and SUZ12 KO used in this study were obtained from the Pasini lab. The the EZH1/2 dKO mESCs were verified via Sanger Sequencing and the SUZ12 KO mESCs were genotyped in the original publications.

#### Bacterial strains

BL21 (DE3) pLysS were grown in 2xTY medium, supplemented with selective antibiotics, at 37 °C.

### METHOD DETAILS

#### Cloning, expression, and purification of PRC2

PRC2 was cloned, expressed, and purified as previously described.^8,16^ Briefly, full-length sequences of EZH2 isoform 2, EED, RBAP48, strep-tagged AEBP2 and residues 80–685 of SUZ12 (residues 80–685) were cloned into the MacroBac system for baculovirus expression in HighFive insect cells.^48^ For experiments involving the subunit JARID2, residues 119–450 (excluding the methylated K116 residue) of JARID2 were also included in the MacroBac plasmid. Expression of PRC2 occurred for 72 hours at 27 °C and pellets were stored at −80 °C until use. All purification steps were performed at 4°C. Pellets were resuspended in 25 mM HEPES, pH 7.9, 250 mM NaCl, 5% glycerol, 0.1% NP-40, 1 mM TCEP, supplemented with 10 μM leupeptin, 0.2 mM PMSF, protease inhibitor cocktail (Roche) and benzonase (Sigma-Aldrich). Cells were lysed by sonication and cleared by centrifugation at 35 000g for 45 minutes. The supernatant was incubated with Step-Tactin Superflow Plus resin (Qiagen) for 6 hours and then washed with low (25 mM HEPES, pH 7.9, 150 mM NaCl, 1 mM TCEP, 5% glycerol, 0.01% NP40) and high salt buffers (25 mM HEPES, pH 7.9, 1 M NaCl, 1 mM TCEP, 5% glycerol, 0.01% NP40) followed by elution with 10 mM desthiobiotin.

The eluate was pooled and incubated with TEV protease over night to cleave off the affinity tag, followed by size exclusion chromatography using a Superose 6 3.2/300 column (Cytiva) equilibrated with 25 mM HEPES pH 7.9, 150 mM NaCl, 2 mM MgCl2, 10% glycerol, and 1 mM TCEP. Purified complex was flash frozen in liquid nitrogen and stored at −80 °C as single-use aliquots.

For the HMTase activity assays, the EZH2 and SUZ12 mutations required for the PRC2 mutants were introduced by site directed mutagenesis, and the subunits were assembled into multi-gene plasmids for baculoviral expression using the GoldenBac assembly protocol.^49^ These mutants were expressed in *T. ni* insect cells (Expression Systems) and purified as described above.

#### Nucleosome purification

Xenopus histones (H2A, H2B, H3, and H4) were expressed and purified as described previously.^32^ The nucleosomal DNA contains a CpG Island sequence and a 5′ biotin tag and was assembled by large scale PCR, purified over an anion exchange column, and further purified by ethanol precipitation. The 226-base pair (bp) nucleosome DNA sequence used for all the studies with Xenopus nucleosomes was 5′ (biotin) CACGCGACTGTGTGCCCGTCAGACGCTGCGCTGCCGGCGGctggagaatcccggtgccgaggccgctcaattggtcgtagacagctctagcaccgcttaaacgcacgtacgcgctgtcccccgcgttttaaccgccaaggggattactccctagtctccaggcacgtgtcagatatatacatcctgtatgcatgc atatcattcgatcggagctcccgatcgatgc - 3′. The CG-rich sequence used is capitalized and the 601-nucleosome positioning sequence^50^ is underlined. For nucleosome assembly, equimolar amounts of all histones were dialyzed into histone refolding buffer (2 M NaCl, 10 mM TRIS, 5 mM EDTA), and the octamer was purified using a Superdex 200 10/300 size exclusion column (Cytiva). The DNA and octamer were mixed in a 1:1.1 ratio and purified over a BioRad prep cell after overnight gradient salt dialysis, as described previously.^32^

To create H1-containing nucleosomes, His-tagged *Xenopus* histone H1.0 and His-tagged murine nuclear assembly factor 1 (mNAP1) were cloned into and recombinantly expressed in BL21-DE3 *E. coli* and purified as follows: For H1, bacterial cell pellets were resuspended in lysis buffer (1 M NaCl, 20 mM Tris pH 7.4, 10 mM imidazole, 10% glycerol, 0.5 mM TCEP) supplemented with DNAse, PMSF and EDTA free protease inhibitor (Roche), before lysis by sonication. After an addition of 1% (v/v) Triton X-100 and centrifugation at 35 000 g for 30 minutes at 4°C, the clarified lysate was incubated on charged Nickel beads (Qiagen) that have been pre-equilibrated in lysis buffer containing 1% (v/v) Triton X-100. Beads were washed with 5 column volumes (CV) of lysis buffer, followed by 5 CV of wash buffer (500 mM NaCl, 20 mM Tris pH 7.4, 10 mM imidazole, 0.5 mM TCEP) until no more protein eluted as monitored by Bradford reagent. H1 was eluted with elution buffer (500 mM NaCl, 20 mM Tris pH 7.4, 500 mM imidazole, 0.5 mM TCEP) and the peak fraction collected for dialysis into 200 mM NaCl, 20 mM HEPES pH 7.4, 0.5 mM TCEP at 4°C over-night. Dialyzed protein was subjected to cation exchange chromatography using a Mono S 5/50 GL column (Cytiva) and subjected to a salt gradient to 1 M NaCl. H1 containing fractions were pooled, frozen in liquid nitrogen and stored at −80° C until further use.

For NAP1, purification was essentially carried out the same as for H1 except for following steps: no Triton X-100 was added during purification and ion exchange chromatography was carried out as anion exchange chromatography and therefore a Mono Q 5/50 GL column was used (Cytiva). Consequently, the ion exchange buffer and the preceding dialysis buffer contained 20 mM Tris pH 7.4 instead of 20 mM HEPES.

NAP1 mediated H1 deposition on nucleosomes was carried out as described previously.^51^ H1 and NAP1 were mixed in a 1:2 molar ratio and incubated at 30 C for 30 minutes in 100 mM NaCl, 20 mM Tris pH 7.4, 0.5 mM EDTA, 10 % glycerol, 1 mM DTT. NAP1-H1 complexes were then incubated with biotinylated nucleosome in a 5 Molar excess for 30 minutes at room temperature. The sample was then directly used for cryo-EM experiments as described below. Excess NAP1-H1 was washed away from biotinylated nucleosomes during cryo-EM sample preparation using streptavidin affinity grids.

#### Negative stain EM

Negative stain analysis of PRC2 was carried out essentially as described before.^8^ Briefly, 4 μl of 200 nM PRC2 were incubated on a continuous carbon grid (EMS) for 45 sec, followed by five successive short incubation steps with 2% (wt/vol) uranyl formate. Excess stain was removed by blotting with filter paper and the grids were dried. Screening and data collection was done using a Talos L120C (Thermo Fisher Scientific) and EPU for automated data acquisition, at an electron dose of 25 e/Å^2^ and a nominal pixel size of 2.44 Å/px. Data processing was done in cryosparc,^36^ CTF estimation was done using CTFFIND4,^52^ and particle picking using the blob picker in cryosparc. For WT PRC2, PRC2^amKR^ and PRC2^Δdim^, 117,434, 248,000 and 176,329 particle images were extracted based on initial picks from and 242, 398 and 286 manually curated micrographs, respectively. Several rounds of 2D classification led to subsets of classes with typical structural features of PRC2, which were overall comparable between WT PRC2 and PRC2^amKR^ and PRC2^Δdim^ mutants. Representative, typical views of intact PRC2 were chosen and adjusted for PRC2 orientation.

#### Cryo-EM grid preparation

To prevent damage of PRC2 by interactions with the air water interface we used streptavidin affinity grids manufactured in-house, as described previously.^16,53,54^

All PRC2-nucleosome complexes were assembled by incubating 200 nM biotinylated nucleosome (containing or lacking H1) with 800 nM PRC2 and 100 μM SAH in 25 mM HEPES pH 7.9, 50 mM KCl, 1 mM TCEP for 30 minutes at RT. 4 μl of the complex were incubated on rehydrated Quantifoil Au 2/2 grids containing the streptavidin affinity layer and incubated for 5 minutes in a humidified chamber. The grid was then washed with two times 10 μl of buffer containing 25 mM HEPES pH 7.9, 50 mM KCl, 1 mM TCEP, 4% Trehalose, and 0.01% NP40. Excess buffer was wicked away with filter paper before adding an additional 2.5 μl of the same buffer. After transfer of the grid into a TF Mark IV Vitrobot the grid was manually blotted for 2–3 s at 18 °C and 100% before plunging it into liquid ethane.

#### Data collection and processing

For the PRC2 dimer, two datasets (dataset 1 and 2) were collected on a FEI Titan Krios G2 cryo-electron microscope operating at 300 kV, equipped with a GIF quantum energy filter and a GATAN K2 direct electron detector in super resolution mode. 3,894 micrographs were collected for dataset 1 and 4,062 micrographs were collected for dataset 2. For each exposure a total of 40 frames were collected with a total dose of 50 e^−/^Å^2^ at a super resolution pixel size of 0.575 Å/pix while varying the defocus between −1.5 and −3.5 μm. Movies were motion corrected and dose weighted using MotionCor2^33^ before subtraction of the streptavidin lattice using in-house MATLAB scripts.^55^ ~600k particles were picked using the convolutional neural network picker implemented in EMAN2.^35^ CTF estimation, particle extraction and initial rounds of 2D and 3D classification were carried out in Relion 3.0^34^ for initial clean-up of the particle stack. After merging particles from both datasets and another round of 3D classification a class corresponding to the PRC2 dimer bound to the nucleosome and a class corresponding to the PRC2 monomer bound to the nucleosome became apparent. Particles were transferred to Cryosparc v4.0^36^ for all further processing. The dimer and the monomer classes were refined independently to resolutions of 6.2 and 4.1 Å, respectively, determined according to the gold-standard FSC = 0.143 criterion.^56,57^ To overcome continuous flexibility inherent to the complexes we used 3DFlex as implemented in Cryosparc to improve the quality of our maps.^19^ Dividing the PRC2 dimer bound to nucleosome into three bodies, where both PRC2 protomers are attached to the nucleosome, and using 5 latent dimensions during the program training phase, revealed several modes of relative motion in the complex and improved the quality of the distal PRC2 protomer (Figure S4). Focused refinements with search parameters adjusted for large movements yielded the final maps which were then filtered by local resolution using manually adjusted B-factors to prevent over-sharpening. The final maps are represented in Figure S2.

For PRC2_J119–450_-H1-Nucleosome, 14,470 movies (dataset 3) were collected on a FEI Titan Krios G2 cryo-electron microscope operating at 300 kV, equipped with a GIF quantum energy filter and a GATAN K3 direct electron detector in super resolution mode. For each exposure a total of 50 frames were collected with a total dose of 50 e^−/^Å^2^ at a super resolution pixel size of 0.575 Å/pix while varying the defocus between −0.8 and −2.5 μm. After motion correction and streptavidin lattice subtraction, 3,658,724 particles were picked using Cryolo. CTF estimation, particle extraction and initial rounds of 2D and 3D classification were carried out in Relion 3.1 for initial clean-up of the particle stack. After another round of 3D classification, a class corresponding to the PRC2_J119–450_ bound to the H1-nucleosome became apparent. Particles were transferred to Cryosparc v4.0 and the map refined to a resolution of 4 Å, determined according to the gold-standard FSC = 0.143 criterion. Local refinements of the H1-nucleosome and PRC2_J119–450_ yielded the final maps with resolutions of 3.6 Å each which were then filtered by local resolution. For the H1.0-nucleosome complex, 2,236 micrographs were collected on a Talos Arctica electron microscope operating at 200kV and equipped with a Gatan K3 direct electron detector using a final pixelsize of 1.14 Å/pix. After motion correction, streptavidin lattice subtraction and CTF estimation data was processed in Cryosparc v4.0 using a standard workflow. A final particle set of 44,742 yielded a reconstruction with a resolution of 3.14 Å. Data was further processed by using 3DFlex to improve regions of the map suffering from flexibility.

#### Model building and visualization

To obtain a model for the allosteric PRC2 dimer and the PRC2 monomer we used a trimmed model of PRC2 bound to an ubiquitylated nucleosome (PDB: 6WKR^16^) as a starting point to perform flexible fitting using Isolde v1.5 in UCSF ChimeraX v1.5^37,58^ into locally refined maps and model building in Coot,^38^ applying appropriate model restraints. Nucleosomal linker DNA was modeled using ChimeraX and then also flexibly fitted into the density using Isolde v1.5. The automethylation loop was modeled using a fragment of JARID2 present in the input structure and also fitted using Isolde v1.5 and Coot.

The same strategy was used to obtain a model for the monomeric PRC2 bound to nucleosome and for PRC2_J119–450_ bound to H1-nucleosome. For the H1 containing nucleosome, PDB: 3NL0 was used as a starting model. All models were then subjected to real space refinement in phenix v1.2,^39^ enabling local grid search, global minimization and ADP refinement, with Ramachandran restraints enabled but secondary structure restraints disabled. For modeling of the automethylation loop, the visually most likely sequence was modeled into the density (with EZH2 K514 being recognized by EED) and refined as described. To remove author bias, all am-loop residues except trimethylated lysine were then mutated to alanine in Coot, renamed to UNK and subjected to another round of ADP-only refinement in phenix. All final models were created by combining the local models into a composite model and refining the final models against the respective consensus reconstruction with model restraints enabled. Refinement parameters and model validation parameters are reported in Table S1.

ChimeraX v1.5^37^ was used to visualize maps and models.

#### Histone methyltransferase (HMTase) assay

To perform the HMTase assay, reactions were carried out in a total volume of 12 μL containing 200 nM each of nucleosome and PRC2 in a reaction buffer (25 mM HEPES pH 7.9, 50 mM NaCl, 2.5 mM MgCl2, 0.25 mM EDTA, 5% glycerol, 1 mM DTT and 80 μM SAM). Where PRC2^CXC^ and its variants were used as potential allosteric activators, the reaction mix including 400 nM PRC2^CXC^ was incubated with 80 μM SAM at room temperature for 1 hour to facilitate automethylation. Subsequently, catalytically active PRC2 and substrate nucleosomes were added. In experiments where the EED allosteric activation was disrupted, 2 μM of the EED inhibitor MAK583 (Selleck Chemicals, S8983)^22^ was included in the reaction. When methyl-lysine containing peptides were used, these were added at the indicated concentrations. For peptide stimulation, synthesized peptides (Genscript) corresponding to aa17–38 of histone H3 (RKQLATKAARSAPATGGVKKPH) methylated at K27, or peptides corresponding to human EZH2 aa502–521 (RLWAAHCRKIQLKKDGSSNH) either non-methylated or trimethylated at K510, K514 or K515 were used. The reaction proceeded at room temperature for 90 minutes and was quenched by the addition of 5x loading buffer and heat inactivation at 95°C for 5 minutes. In case of peptide stimulation, the peptides were added immediately after the nucleosome. Separation by gel electrophoresis was performed with 4–20% Mini-PROTEAN^®^ TGX^™^ precast protein gels (BioRad) and the stain-free signal was detected according to the manufacturer’s instructions. Proteins were subsequently transferred to a 0.2 μM PVDF membrane at 90V for 10 min and 60 V for 30 min. The membranes were probed with antibodies against H3K27me3 (Active Motif, 39155), H3K27me2 (Cell Signaling, D18C8) and H4 (Cell Signaling, L64C1). Reactions were performed in multiplets and detected with a ChemiDoc MP (BioRad). Densitometric analysis was performed using Image Lab Software version 6.1.0 (BioRad) by background-correcting the signal to the negative control and normalizing it against the WT signal. GraphPad Prism was used for visualization. Every experiment was performed at least three times independently.

#### Electrophoretic mobility shift assay (EMSA)

EMSA was performed using a 5% native TBE gel in 0.2x TBE buffer with a total volume of 15 μL reactions of 50 nM nucleosome and increasing concentrations of PRC2 in triplicates in binding buffer (25 mM HEPES pH 7.9, 50 mM NaCl, 1 mM DTT and 100 μM SAH). The reaction mixture was incubated at room temperature for 30 minutes to allow for binding. The gels were then stained with SYBR^™^ Gold (Thermo Fisher) according to the manufacturer’s instructions. The stained gels were imaged using a ChemiDoc MP (BioRad) imager, and densitometric analysis was performed using Image Lab Software version 6.1.0 (BioRad). The bands of the shifted (bound) and free nucleosomes (unbound) were identified and boxed out. After background correction, the bound signal was divided by the sum of both signals to determine the bound fraction.

#### Murine embryonic stem cell cultivation and differentiation

The EZH1/2 dKO mESCs (strain of origin E14TG2a) and SU12 KO used in this study were obtained from the Pasini lab and previously characterized.^25,26^ The cells were cultured on 0.1% gelatin-coated dishes in mESC media consisting of GMEM (Gibco) supplemented with 20% ES-grade fetal bovine serum (Gibco), 2 mM glutamine (Gibco), 100 U/ml penicillin, 0.1 mg/ml streptomycin (Gibco), 0.1 mM non-essential amino acids (Gibco), 1 mM sodium pyruvate (Gibco), 50 μM ß-mercaptoethanol (Gibco), 1000 U/ml ESGRO Leukemia Inhibitory Factor (LIF, Sigma Aldrich, ESG1107), and GSK3β and MEK 1/2 inhibitors (Axon Medchem BV) to a final concentration of 3 μM and 1 μM, respectively. For maintaining a confluency of between 60 and 70%, cells were passaged every 2–3 days by washing twice with phosphate-buffered saline (PBS) and dissociation with 0.25% Trypsin (Life Technologies, 25200056).

For transfection, EZH2 wild type and EZH2amKR were cloned into a pPB_PGK plasmid and co-transfected with a piggyback transposase using Lipofectamine 2000 (Thermo Fisher Scientific) following the manufacturer’s instructions and were selected with puromycin (1 μg/ml).

For differentiation mESCs were seeded at a density of 10500 cells/cm^2^ in mESC media lacking LIF, GSK3β, and MEK 1/2 for 12 hours to allow for cell attachment. The media was then exchanged and supplemented with 0.1 μM all-trans-retinoic acid, and subsequently changed every 48 hours.

#### Whole cell lysis and western blotting

Total protein lysis was performed by incubating the cells on ice for 30 minutes followed by sonication in ice-cold RIPA buffer (50 mM Tris-HCl pH 7.4, 150 mM NaCl, 1 mM EDTA, 1 % NP-40, 1% Na-deoxycholate, 0.1 % SDS) supplemented with protease inhibitors and 1 μg/mL Benzonase (produced in-house). Protein concentration was determined with Pierce^™^ Rapid Gold BCA Protein-Assay-Kit (Thermo Fisher) and normalized to 60 μg before being supplemented with Laemmli sample buffer. Protein lysates were separated via SDS-PAGE and transferred to PVDF membrane at 90V for 120 minutes. The membranes were probed with antibodies against H3K27me3 (ActiveMotif, 39155), SUZ12 (Cell Signaling, 3737S), EZH2 (Cell Signaling, 3147S), H4 (Cell Signaling, L64C1), and β-Actin (Sigma-Aldrich, A5441-.2M). The proteins were detected with a ChemiDoc MP (BioRad)

#### RNA isolation and RNA seq

Total RNA was isolated from cells using NucleoSpin RNA Kit (MACHEREY NAGEL, cat. no. 740955) according to manufacturer’s protocol. Libraries for RNA seq were generated using QuantSeq 3’ mRNA Seq Library Prep Kit FWD with Unique Dual Indices for Illumina (Lexogen, cat. no 115.384). Sequencing was performed on an Illumina NovaSeq 6000 platform with NovaSeq 6000 SP Reagent Kit v1.5 100 cycles (Illumina, cat. no. 20028401). RNA seq experiments were conducted with three independent biological replicates.

#### RNA seq data analysis

Upon quality trimming using bbduk^42^ (k=13 ktrim=r useshortkmers=t mink=5 qtrim=t trimq=10 minlength=20), fastq files were aligned with STAR^43^ v2.7.3a to the *mm10* mouse reference genome. BAM files were down sampled to 8 million reads with samtools^44^ v1.13 and counted by HTSeq^45^ v2.0.1 (-m union -s no -t exon). Differential expression analysis was performed using DESeq2^46^ v1.42.3 either in multifactorial (including all timepoints for marker gene visualization and PCA) or unifactorial mode (focus on terminally differentiated cells for MA plots and GSEA). Expression of marker genes during RA-driven differentiation were extracted from DESeq2 (counts(dds, normalization = TRUE)) and plotted with ggplot2 v3.5.0. PCA with mean log transformed DESeq2 (rlogTransformation(dds, blind = FALSE)) was plotted with the ggplot2. MA plots were generated directly from DESeq2 results. Pre-ranking of RNA-seq data for GSEA was conducted based on significance (adjusted p-value) and log2foldchange. GSEA was run with GSEA software v4.3.3^47^ with 1,000 permutations against h.all.v2023.2.Hs.symbols.gmt for max size = 3,000 and min size = 10. Extracted NES for significantly altered pathways (NOM p-value <=0.1) were plotted with ggplot2 as bar plots. Overlapping significantly altered pathways between genotypes were plotted as Venn diagrams with VennDiagram v1.7.3.

#### Molecular dynamics

All-atom simulations were performed with the GROMACS 2021 MD package^41^ using the EZH2 SET domain (residues 490–751 of EZH2 isoform 2) and the nucleosome from PDB: 6WKR as a starting model. Simulations encompassed unmethylated or trimethylated lysines in positions 510, 514 and 515 of the SET domain, either in the presence or absence of the nucleosome, in 100 mM KCl, with two replicates for each case. The parameters for the modified lysines were taken from,^59^ *while Amber forcefields*^60,61^ were used for protein, DNA, and ions. For water molecules, the TIP3P model^62^ was used. Long-range electrostatics were evaluated with particle-mesh Ewald summation,^63^ and all hydrogen bonds were constrained with the LINCS algorithm.^40^ A leap-frog integrator was considered with a 2 fs timestep, and a 1.2 nm cutoff was used for both the electrostatic and Van der Waals interactions. All simulations underwent an initial energy minimization with the steepest descent method,^64^ followed by a 50 ns NpT equilibration with a Parrinello-Rahman barostat^65^ at 1 atm and Nose-Hoover thermostat^66^ at 300 K. Position restraints were applied to the phosphorus atoms of the nucleosomal DNA as well as to the first and last five amino acids of the EZH2 SET domain. 1000 ns simulations were performed for all cases with the position restraints in place. The number of contacts between bridge-helix (EZH2 residues 501–617) and the H3 histone tail were calculated using GROMACS inbuilt routines. For the helicity analysis, the change % in helicity for the bridge-helix was calculated using GROMACS at an interval of 50 ns for all the cases.

#### Mass spectrometry

##### Mass spectrometric analysis of recombinant PRC2 used for cryo-EM

To analyze determine the number of methylated lysine residues on the automethylation loop, ~150 μg of PRC2 were first unfolded and reduced in fresh 6.4 M urea and 10 mM DTT and incubated at 55 °C for 20 minutes. Cysteines were alkylated using 20 mM iodoacetamide, followed by incubation at RT for 30 min in the dark and subsequent quenching with an additional 30 mM DTT. Quenching was allowed to occur for 20 min at RT before dialysis at 4 °C against 50 mM Tris pH 7.7, 5 mM CaCl2, 2 mM EDTA and 5 mM DTT to remove urea and iodoacetamide. The sample was then digested with 500 ng Arg-C endopeptidase overnight at RT. The reaction was stopped by incubation for 10 minutes at 95 °C. Liquid chromatography – mass spectrometry measurements of the sample were performed in the QB3/Chemistry Mass Spectrometry Facility at UC Berkeley as described elsewhere.^67^ Briefly, digested PRC2 was analyzed using a Synapt G2-Si ion mobility mass spectrometer equipped with a nanoelectrospray ionization source (Waters) in line with an ACQUITY M-class ultraperformance LC system.

Raw data acquisition was controlled using MassLynx software (version 4.1), and peptide identification and relative quantification were performed using Progenesis QI for Proteomics software (version 4.0; Waters). Calculation of the percentage of lysine methylation (mono-, di-, tri-, or unmethylated) was performed by dividing the abundance of a peptide bearing one or several modifications by the total abundance and multiplying by 100.

##### Mass spectrometry of am-loop methylation of PRC2^CXC^ and PRC2^CXC/Δdim^

Sample preparation for LC/MS/MS is based on the SP3 protocol.^68^ 15 μg protein extracts were taken up in 100 μL 1× SP3 lysis buffer (final concentrations: 1% (wt/vol) SDS, 10 mM TCEP, 200 μL 40 mM chloracetamide, 250 mM HEPES pH 8) and heated for 5 min at 90 °C. Since the samples were purified proteins the Benzonase treatment was skipped. Next samples were mixed with 75 μg hydrophobic (#65152105050250) and 75 μg hydrophilic (#45152105050250) SeraMag Speed Beads (Cytiva) (bead to protein ratio 10 to 1) and gently mixed. Then 100 μL 100% vol/vol Ethanol (EtOH) was added before incubation for 20 min at 24°C shaking vigorously. The beads were collected on a magnet and the supernatant aspirated. The beads were then washed 4 times with 180 μL 80 % EtOH (collection time on the magnet minimum of 4 min). The beads were finally taken up in Arg-C incubation buffer (50 mM Tris-HCl (pH 7.6–7.9), 5 mM CaCl_2_, 2 mM EDTA) containg 150 ng ArgC (Promega V1881; activated in Arg-C activation buffer (50 mM Tris-HCl (pH 7.6–7.9), 50 mM DTT, 2 mM EDTA). To help bead dissociation, samples were incubated for 5 min in a sonification bath (pre-heated to 37°C). Samples were incubated overnight, shaking vigorously (1300 rpm). Next day samples were acidified with formic acid (FA, final 1% vol/vol) before collection on a magnet. The supernatants were transferred to a fresh Eppendorf tube, before removing trace beads using a magnet for 5 min. The tryptic digests were then desalted on home-made C18 StageTips as described.^69^ Briefly, peptides were immobilized and washed on a homemade 2 disc C18 StageTip. Samples were then dried using a vacuum concentrator (Eppendorf) and the peptides were taken up in 0.1% formic acid solution (10 μL) and directly used for LC-MS/MS experiments.

LC-MS/MS experiments were performed on an Orbitrap Fusion Lumos (Thermo) that was coupled to an Vanquish Neo liquid chromatography (LC) system (Thermo). The LC was operated in the one-column mode. The analytical column was a fused silica capillary (75 μm × 28 cm) with an integrated frit emitter (CoAnn Technologies) packed in-house with Kinetex C18-XB core shell 1.7 μm resin (Phenomenex). The analytical column was encased by a column oven (Sonation) and attached to a nanospray flex ion source (Thermo). The column oven temperature was adjusted to 50 °C during data acquisition. The LC was equipped with two mobile phases: solvent A (0.2% formic acid, FA, 99.9% H_2_O) and solvent B (0.2% formic acid, FA, 80% Acetonitrile, ACN, 19.8% H_2_O). All solvents were of UPLC grade (Honeywell). Peptides were directly loaded onto the analytical column with a maximum flow rate that would not exceed the set pressure limit of 950 bar (usually around 0.5 μL/min). Peptides were subsequently separated on the analytical column by running a 105 min gradient of solvent A and solvent B (start with 8% B; gradient 8% to 80% B for 80:00 min; gradient 80% to 100% B for 19:00 min and 100% B for 6:00 min) at a flow rate of 250 nl/min. The mass spectrometer was operated using Tune v3.5.3881.18. The mass spectrometer was set in the positive ion mode. Precursor ion scanning was performed in the Orbitrap analyzer (FTMS; Fourier Transform Mass Spectrometry) in the scan range of m/z 380–1800 and at a resolution of 120000 with the internal lock mass option turned on (lock mass was 445.120025 m/z, polysiloxane).^70^ Product ion spectra were recorded in a data dependent fashion in the FTMS at a resolution of 30000. The ionization potential (spray voltage) was set to 2.3 kV and ion transfer capillary temperature was set to 270 °C. RF-lens was set to 40% to facilitate the transition of larger peptides. Peptides were analyzed using a repeating cycle (cycle time max 3 sec) consisting of a full precursor ion scan (AGC standard; acquisition time “auto”) followed by a variable number of product ion scans (AGC 200% and acquisition time 70 ms) where peptides are isolated based on their intensity in the full survey scan (threshold of 50000 counts) for tandem mass spectrum (MS2) generation that permits peptide sequencing and identification. Fragmentation was achieved by stepped Higher Energy Collision Dissociation (sHCD) (NCE 25, 02, 40). During MS2 data acquisition dynamic ion exclusion was set to 30 seconds and a repeat count of one. Ion injection time prediction, preview mode for the FTMS, monoisotopic precursor selection and charge state screening were enabled. Only charge states between +3 and +10 were considered for fragmentation.

###### Data analysis.

RAW spectra were analysed in Proteome Discoverer (v2.5.0.400) using Sequest HT and MS Amanda 2.0 Nodes. The MS/MS spectra data were searched against a project specific database containing the 5 sequences of the investigated protein complex (ACE_0861_SOI_v01.fasta; 5 entries). All searches also included a contaminants database search (contaminants.fasta, 245 entries). The contaminants database contains known MS contaminants and was included to estimate the level of contamination. The searches allowed oxidation of methionine residues (16 Da), Monomethylation (14 Da)/Dimethylation (28 Da)/Trimethylation (42 Da) on Lysin as variable modifications. Carbamidomethylation on Cystein (57) was selected as static modification. Enzyme specificity was set to “Trypsin_R (full)” (which is basically the specificity of “ArgC”. The precursor mass tolerance was set to ±10 ppm. The MS/MS match tolerance was set to ±0.02 Da. The PSM validation was done using the Percolator Node (target FDR strict 0.01 and relaxed 0.05 based on target-decoy approach). Feature detection was done using the Minora Node (default settings). In the consensus workflow the Feature Mapper node was set to align retention times (max window 10 min). The “precursor ion quantification” node was set to do ion quantification based on intensity. The calculated abundances for the combined EP01 (PRC2^CXC^) and EP02 (PRC2^CXC/Δdim^) runs were extracted from the peptide groups output together with the coefficients of variation of the variation. All values were transferred to the log_2_(x) scale and used to generate the bar diagram.

### QUANTIFICATION AND STATISTICAL ANALYSIS

Statistical analysis of EMSA and HMTase activity assays was performed by GraphPad Prism 10, representing the SD and SEM of n=3–6 replicates as described in the legend of Figure S6 and STAR Methods, statistical significance was calculated using t-test. All biochemical assays were done in replicates of n = 3 or more, where each replicate represents independent biological reactions. Mass-spectrometry data presented in Figure S6 show results with SD of four technical replicates. Analysis of helicity by molecular dynamics simulations presented in Figure 5 show SD of two technical replicates as described in the figure legend and STAR Methods. Statistical analysis of RNA-seq data is based on three independent biological replicates and was analyzed as detailed in the STAR Methods and legend of Figure S8. Normalized expression data presented in Figure S8A shows the SD from three independent biological replicates and was analyzed using HTSeq^45^ v2.0.1 and DESeq2^46^ v1.42.3.

## Supplementary Material

Supplemental

## Figures and Tables

**Table:** KEY RESOURCES TABLE

REAGENT or RESOURCE	SOURCE	IDENTIFIER
Antibodies		
Rabbit polyclonal Histone H3K27me3 antibody (pAb)	Active Motif	Cat #39155; RRID: AB_2561020
Rabbit monoclonal Di-Methyl-Histone H3 (Lys27)	Cell Signaling	Cat #9728; RRID: AB_1281338
Mouse monoclonal Histone H4 (L64C1) antibody	Cell Signaling	Cat #2935; RRID: AB_1147658
SUZ12 (D39F6) XP^®^ Rabbit mAb	Cell Signaling	Cat #3737S; RRID: AB_2196850
Ezh2 (AC22) Mouse mAb	Cell Signaling	Cat #3147S; RRID: AB_10694383
Monoclonal Anti-beta-Actin	Sigma-Aldrich	A5441-.2M; RRID: AB_476744
Bacterial and virus strains		
BL21 (DE3) pLysS	Novagen	Cat #70236
DH10 EmBacY	Geneva Biotech	DH10EMBacY
Sf9	Expression Systems	Cat #94-001F
Tni	Expression Systems	Cat #94-002F
Chemicals, peptides, and recombinant proteins		
Step-Tactin Superflow Plus	Qiagen	#30004
glycogen synthase kinase (GSK-3) inhibitor	Axon Medchem	AXON 1386 / CHIR99021 / CT99021 / CAS: 252917-06-9
MEK 1/2 Inhibitor Mirdametinib PD0325901	Axon Medchem	AXON 1408 / PD0325901 / CAS: 391210-10-9
ESGRO^®^ Recombinant Mouse LIF Protein	Sigma Aldrich	ESG1107
Streptavidin	New England Biolabs (NEB)	Cat # N7021S
1,2-dipalmitoyl-sn-glycero-3-phosphoethanolamine-N-(biotinyl) (sodium salt)	Avanti Lipids	Cat # 870285 / CAS: 384835-54-5
EED inhibitor MAK683	Selleck Chem	Cat # S8983 / CAS: 1951408-58-4
SYBR^™^ Gold	Thermo Fisher	S11494
H3_aa17-38_K27me3	Genscript	N/A
EZH2_aa502-521	Genscript	N/A
EZH2_aa502-521_K510me3	Genscript	N/A
EZH2_aa502-521_K514me3	Genscript	N/A
EZH2_aa502-521_K515me3	Genscript	N/A
Streptavidin affinity grids	In house	N/A
ArgC Endopeptidase	Promega	Cat #V1881
S-(5′-Adenosyl)-L-methionine chloride dihydrochloride	Sigma Aldrich	Cat #A7007 / CAS: 86867-01-8
S-(5′-Adenosyl)-L-homocysteine	Sigma Aldrich	Cat # A9384 / CAS: 979-92-0
Critical commercial assays		
NucleoSpin RNA Kit	MACHEREY NAGEL	Cat #740955
NovaSeq 6000 Reagent Kits	Illumina	Cat # 20028401
Deposited data		
PRC2 dimer bound to nucleosome, atomic model	This paper	PDB: 8T9G
PRC2 monomer bound to nucleosome, atomic model	This paper	PDB: 8TAS
PRC2J119-450 bound to H1-nucleosome, atomic model	This paper	PDB: 8TB9
PRC2 dimer bound to nucleosome, cryo-EM density map	This paper	EMDB: EMD-41110
PRC2 monomer bound to nucleosome, cryo-EM density map	This paper	EMDB: EMD-41141
PRC2J119-450 bound to H1-nucleosome, cryo-EM density map	This paper	EMDB: EMD-41146
H1-nucleosome complex, cryo-EM density map	This paper	EMDB: EMD-41147
Cryo-EM raw micrographs	This paper	EMDB: EMPIAR-11607
RNAseq Data	This paper	GEO ID GSE234793
MS proteomics analysis of PRC2 automethylation	This paper	PRIDE ID: PXD051876
Raw gel and immunoblotting images	This paper	Mendeley Data doi: https://doi.org/10.17632/m65ctymnbf.1
Experimental models: Cell lines		
EZH1/2 dKO mESCs	Lavarone et al.^25^	E14TG2a
SUZ12 KO mESCs	Pasini et al.^26^	N/A
EZH1/2 dKO, EV control cells	This paper	N/A
EZH1/2 dKO, WT EZH2 rescue cells	This paper	N/A
EZH1/2 dKO, EZH2^amKR^ rescue cells	This paper	N/A
SUZ12 KO mESCs, EV control cells	This paper	N/A
SUZ12 KO mESCs, WT SUZ12 rescue cells	This paper	N/A
SUZ12 KO mESCs, SUZ12^Δdim^ rescue cells	This paper	N/A
Oligonucleotides		
Recombinant DNA		
pET3d_H2A_Xen_wt	Dyer et al.^32^	N/A
pET3d_H2B_Xen_wt	Dyer et al.^32^	N/A
pET3d_H3_Xen_wt	Dyer et al.^32^	N/A
pET3d_H4_Xen_wt	Dyer et al.^32^	N/A
p438-PRC2_emTwStr	This paper	N/A
p438-PRC2_emTwStr_SUZ12_deltaDim	This paper	N/A
p438-PRC2_emTwStr_EZH2_amKR	This paper	N/A
p438-PRC2_emTwStr_EZH2_CXC	This paper	N/A
p438-PRC2_emTwStr_EZH2_CXC_amKR	This paper	N/A
p438-PRC2_emTwStr_EZH2_CXC_SUZ12_deltaDim	This paper	N/A
pGB-PRC2_emTwStr_EZH2_amKR_EED_Y365A	This paper	N/A
pGB-PRC2_emTwStr_EZH2_amKR	This paper	N/A
Super PiggyBac Transposase Expression Vector	System Biosciences,	Cat # PB200PA-1
pPB_PGK-Puro-T2A-IRES_EV	This paper	N/A
pPB_PGK-Puro-T2A-EZH2	This paper	N/A
pPB_PGK-Puro-T2A-EZH2_amKR	This paper	N/A
pPB_PGK-Puro-T2A-SUZ12	This paper	N/A
pPB_PGK-Puro-T2A-SUZ12_C	This paper	N/A
Software and algorithms		
MotionCor2	Zheng et al.^33^	N/A
Relion 3.0	Zivanov et al.^34^	N/A
EMAN2	Tang et al.^35^	N/A
Cryosparc v4.0	Punjani et al.^36^	N/A
3DFlex	Punjani et al.^19^	N/A
UCSF ChimeraX v1.5	Pettersen et al.^37^	N/A
Coot	Emsley et al.^38^	N/A
phenix v1.2	Adams et al.^39^	N/A
LINCS algorithm	Hess et al.^40^	N/A
GROMACS 2021 MD package	Hess et al.^41^	N/A
Bbduk	Bushnell et al.^42^	N/A
STAR v2.7.3a	Dobin et al.^43^	N/A
samtools v1.13	Li et al.^44^	N/A
HTSeq v2.0.1	Anders et al.^45^	N/A
DESeq2 v1.42.3	Love et al.^46^	N/A
GSEA software v4.3.3	Subramanian et al.^47^	N/A
Image Lab Software 6.1	BioRad	Cat #12012931

## Highlights

- Human PRC2 dimerizes asymmetrically while bound to nucleosomes
- The nucleosome-proximal PRC2 is allosterically activated by the other PRC2 via EED
- Dimerization-driven stimulation is mediated by automethylated EZH2 K510
- Allosteric dimerization affects context-dependent PRC2 function
