# Supplementary material for: Activation of automethylated PRC2 by dimerization on chromatin
Source: Mol Cell. Author manuscript; Available in PMC 2025 Apr 9. (PMC11980035; doi:10.1016/j.molcel.2024.08.025)
Supplement: Supplemental [file NIHMS2071426-supplement-Supplemental.pdf]

Molecular Cell, Volume *84*

Supplemental information

Activation of automethylated PRC2 by dimerization on chromatin

Paul V. Sauer, Egor Pavlenko, Trinity Cookis, Linda C. Zirden, Juliane Renn, Ankush Singhal, Pascal Hunold, Michaela N. Hoehne-Wiechmann, Olivia van Ray, Farnusch Kaschani, Markus Kaiser, Robert Hänsel-Hertsch, Karissa Y. Sanbonmatsu, Eva Nogales, and Simon Poepsel

Activation of automethylated PRC2 by dimerization on chromatin

Supplementary Information

Supplementary Table 1 - Cryo-EM data collection and processing, related to Figures 1-4

**Title**	**PRC2 dimer**	**PRC2^{prox}**	**NCP (dimer)**	**PRC2^{dist}**	**H1-NCP**
**EMD**	**41110**				**41147**
**PDB**	**8T9G**				
Dataset	1+2				3
**Data collection and processing**					
Magnification	43k				43k
Voltage (kV)	300				300
Electron exposure (e–/Å^2)	40				50
Defocus range (μm)	(-1.5) – (-3.5) μm				(-0.8) – (-2.5) μm
Pixel size (Å)	0.575				0.57
Symmetry imposed	C1				C1
Initial particle images (no.)	600 315				374 426
Final particle images (no.)	34 000			18 317	44 742
Map resolution (Å)<br>FSC threshold 0.143	6.2	5.7	4.1	9.1	3.1
Map resolution range (Å)	4-12	4-12	3.5-6	7.5-12	2.8 - 6
**Refinement**					
Initial model used	Composite model	PDB 6WKR	PDB 6WKR	PDB 6WKR	
Model resolution (Å)<br>FSC threshold 0.5	6.7	5.7	4.2	8.8	
Model composition					
Non-hydrogen atoms	87583	15888	14919	15114	
Protein residues	4589	1954	772	1863	
Nucleotide	430		430		
B factors (Å^2)					
Protein	392.51	110.30	52.87	258.98	
Nucleotide	287.95		342.18		
R.m.s. deviations					
Bond lengths (Å) (# > 4σ)	0.005 (0)	0.004 (0)	0.005 (0)	0.003 (0)	
Bond angles (°) (# > 4σ)	0.842 (46)	0.720 (4)	0.681 (1)	0.638 (6)	
Validation					
MolProbity score	2.43	2.63	2.02	2.40	
Clashscore	28.43	38.03	19.31	27.54	
Poor rotamers (%)	0.07	0.00	0.00	0.00	
Ramachandran plot					
Favored (%)	91.91	89.31	96.43	92.50	
Allowed (%)	8.00	10.64	3.57	7.50	
Disallowed (%)	0.09	0.05	0.00	0.00	

Supplementary Table 1 continued - Cryo-EM data collection and processing, related to Figures 1-4

**Title**	**PRC2 monomer**	**PRC2 top lobe**	**NCP (monomer)**	**$PRC_{J119-450}$-H1-NCP**	**$PRC2_{J119-450}$**	**H1-NCP**
**EMD**		**41141**			**41146**	
**PDB**		**8TAS**			**8TB9**	
Dataset		1 + 2			4	
**Data collection and processing**						
Magnification		43k			53k	
Voltage (kV)		300			300	
Electron exposure (e–/Å^2)		40			50	
Defocus range (μm)		(-1.5) – (-3.5) μm			(-0.8) – (-2.5) μm	
Pixel size (Å)		0.575			0.47	
Symmetry imposed		C1			C1	
Initial particle images (no.)		600 315			234 562	
Final particle images (no.)		69 000			55 918	
Map resolution (Å)<br>FSC threshold 0.143	4.1	5.9	3.8	4	3.6	3.6
Map resolution range (Å)	3-12	4.5-10	3-5	3-10	3-8	3-8
**Refinement**						
Initial model used	Composite model	PDB 6WKR	PDB 6WKR	Composite model	PDB 6WKR	PDB 3NL0
Model resolution (Å)<br>FSC threshold 0.5	6.0	5.8	3.8	6.2	3.8	3.8
Model composition						
Non-hydrogen atoms	29458	8680	14137	30242	15685	14514
Protein residues	2683	1073	773	2790	1941	849
Nucleotide	386		394	386		384
B factors (Å^2)						
Protein	74.74	143.40	30.52	312.86	99.77	75.98
Nucleotide	86.27		116.96	312.19		175.94
R.m.s. deviations						
Bond lengths (Å) (# > 4σ)	0.006 (1)	0.003 (0)	0.004 (0)	0.004 (0)	0.008 (3)	0.004 (0)
Bond angles (°) (# > 4σ)	0.695 (1)	0.644 (0)	0.588 (2)	0.677 (3)	0.822 (8)	0.578 (1)
Validation						
MolProbity score	2.21	2.35	1.78	2.26	2.11	1.64
Clashscore	19.04	24.64	13.42	25.04	14.55	8.01
Poor rotamers (%)	0.00	0.00	0.00	0.29	0.46	0.29
Ramachandran plot						
Favored (%)	93.39	92.61	97.23	94.59	93.22	96.75
Allowed (%)	6.50	7.20	2.77	5.34	6.68	3.25
Disallowed (%)	0.11	0.19	0.00	0.07	0.11	0.00

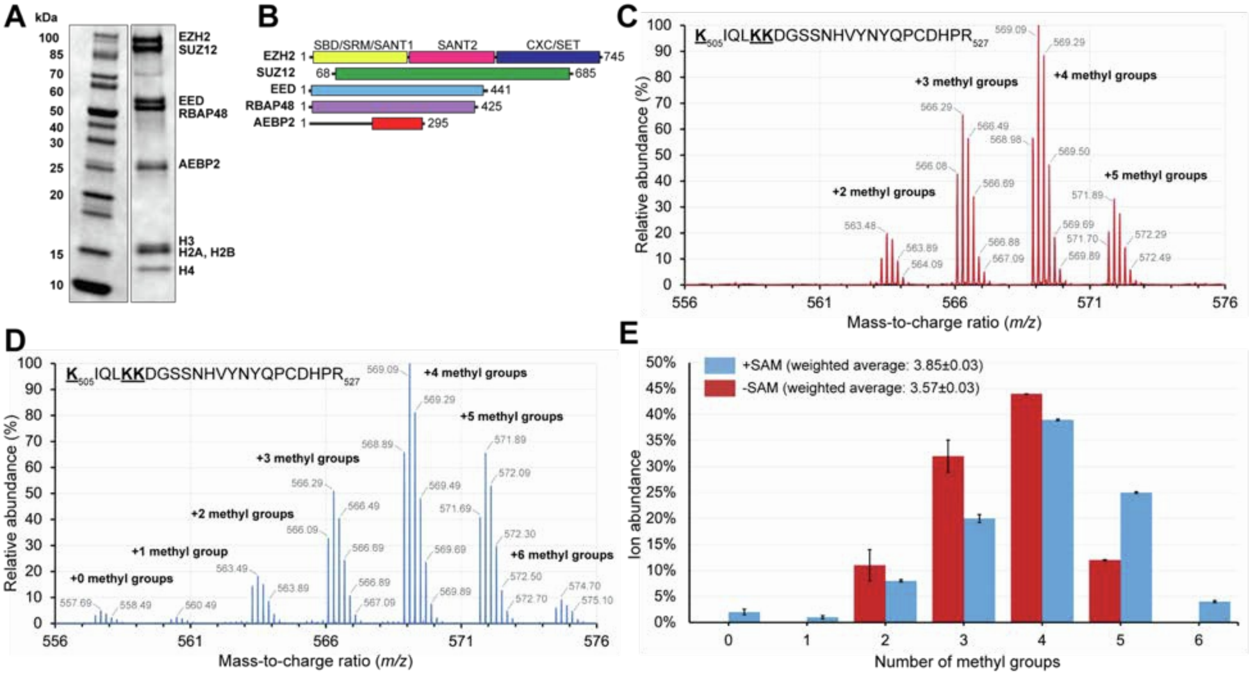

Supplementary Figure 1: PRC2 purification and automethylation, related to Figure 1 and STAR methods.
(A) SDS-PAGE showing subunits of PRC2 and nucleosome used in this study. (B) PRC2 subunits and constructs used in this study. The colors of the domains and subunits are the same as in Fig. 1. (C) Mass spectrometric analysis of automethylated PRC2. Representative high-resolution mass spectra showing detail for the $[M+5H]^{5+}$ ion group for the indicated EZH2 peptide bearing automethylated lysine residues. Cysteines have been alkylated during experiment. (D), like C but after addition of 5mM SAM during protein purification. E, Ion abundance plot showing the number of methyl groups present on average in -SAM and +SAM sample on the automethylation peptide. Error bars show standard deviation (N=2).

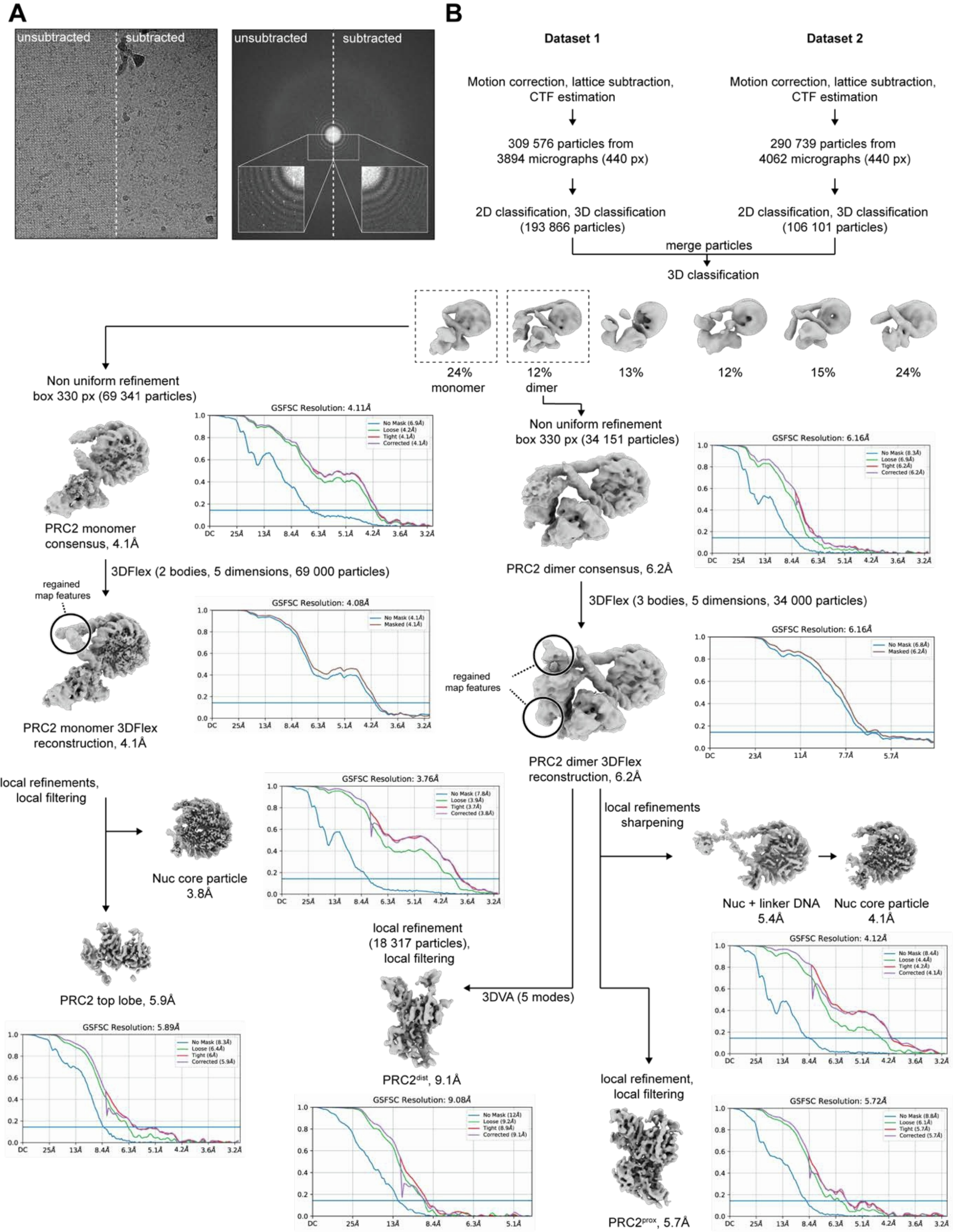

Supplementary Figure 2 - Cryo-EM data processing workflow, related to Figures 1-4. *(A) representative cryo-EM micrograph before and after subtraction of the streptavidin lattice (left) and corresponding Fourier transform showing presence/absence of streptavidin*

diffraction peaks (right). (B) data processing scheme for PRC2 dimer and PRC2 monomer. See Methods for description.

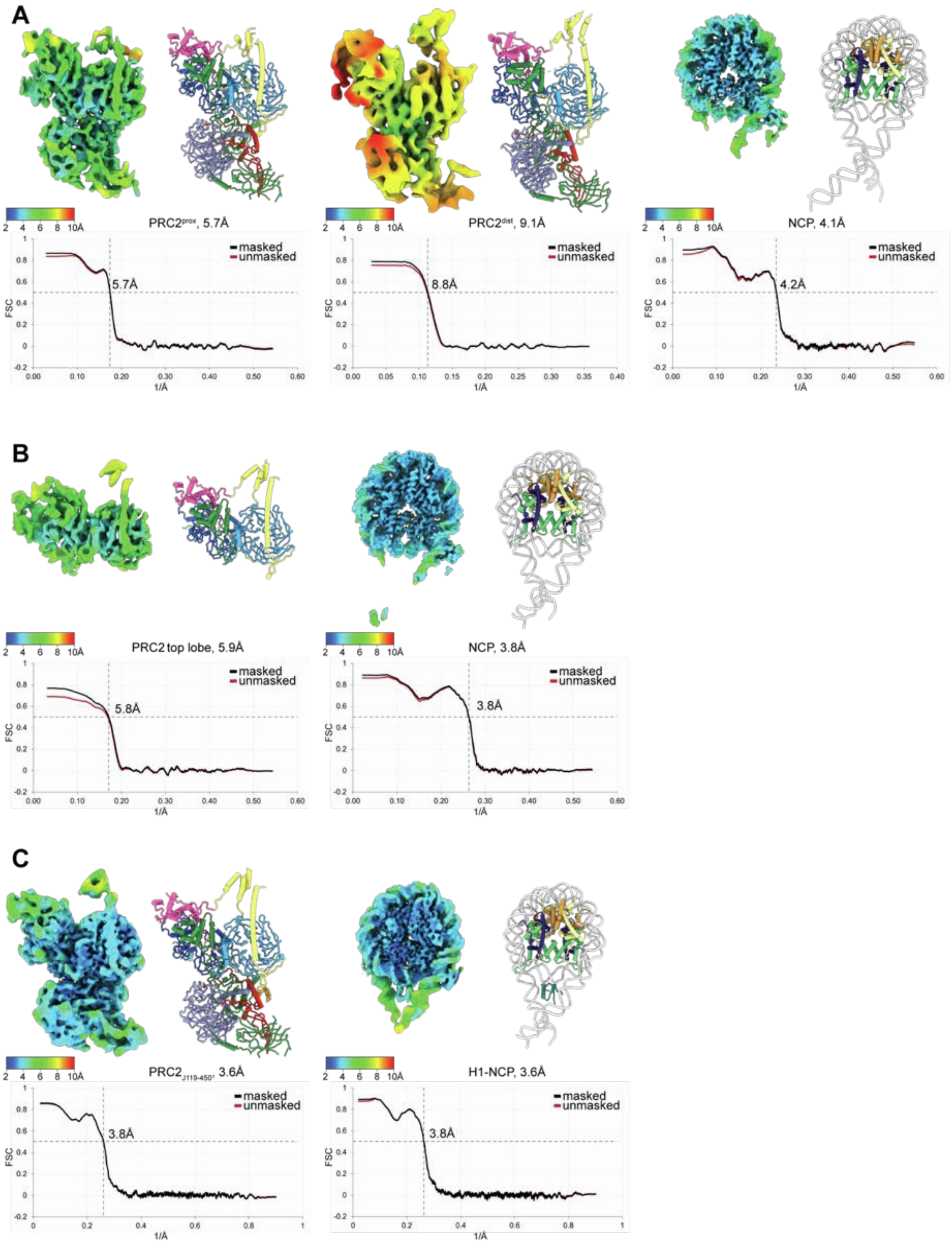

Supplementary Figure 3: Local resolution and model validation, related to Figures 1-4. *Local resolution variation and cartoon representation of the final refined model and masked/unmasked map-to-model Fourier shell correlation for (A) PRC2 dimer, (B) PRC2 monomer, (C) H1-nucleosome-PRC2*$_{J119\text{-}450}$*. For each model, the resolution of the cryo-EM model at FSC = 0.143 is provided, while the map-to-model FSC plots show the masked resolution at FSC = 0.5.*

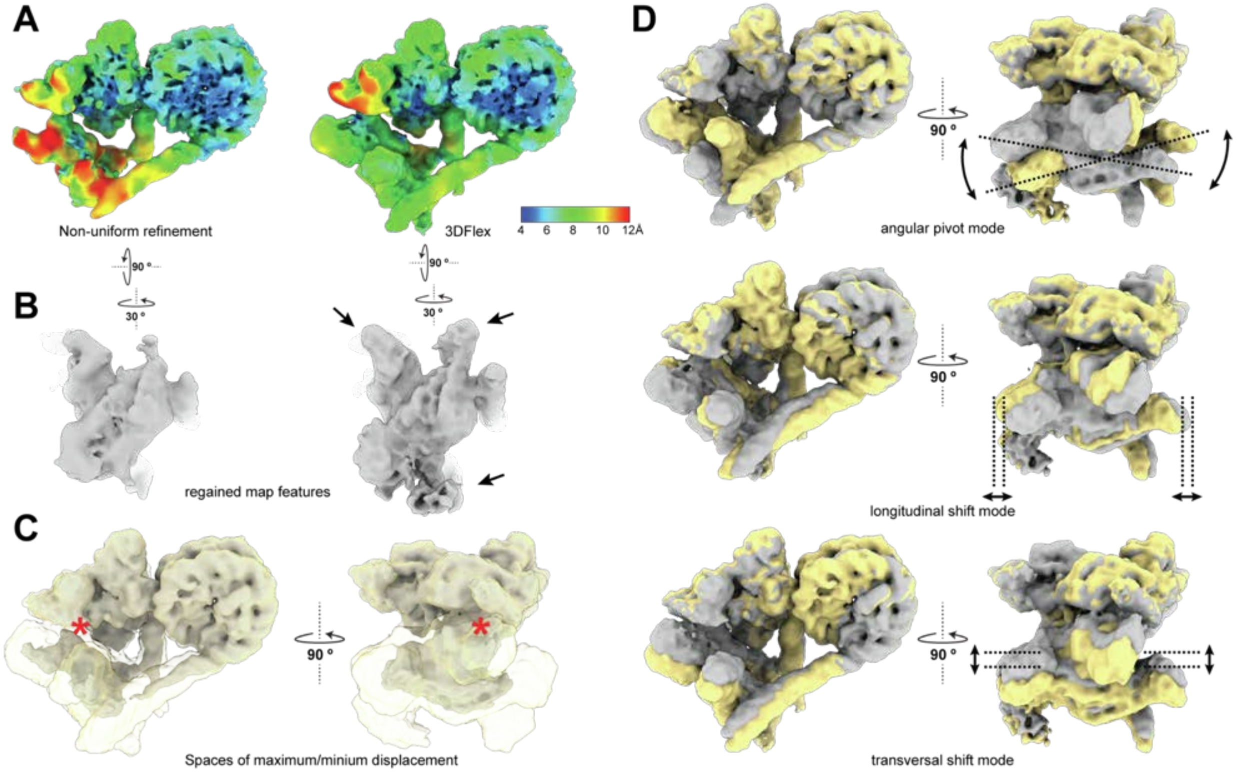

Supplementary Figure 4: PRC2^{dist} displays large amount of continuous flexibility, related to Figures 1-4.

(A) Local resolution before (left) and after (right) 3DFlex analysis, showing gain in resolution at the flexible distal PRC2. (B) Comparison of map features at comparable threshold of PRC2^{dist} before (left) and after (right) 3DFlex analysis. Arrows point to parts of the map where the quality has visibly improved. (C) Visualization of continuous flexibility of PRC2 dimer. Yellow, transparent map shows the extent of space sampled by the complex as calculated with 3DFlex.[19] Grey, solid map shows the region of least variability where density is always present. Red asterisk marks the position of the automethylation loop within the less variable region. (D) Three example modes of movement of the PRC2 dimer, showing pivoting and shifting motions of the distal PRC2.

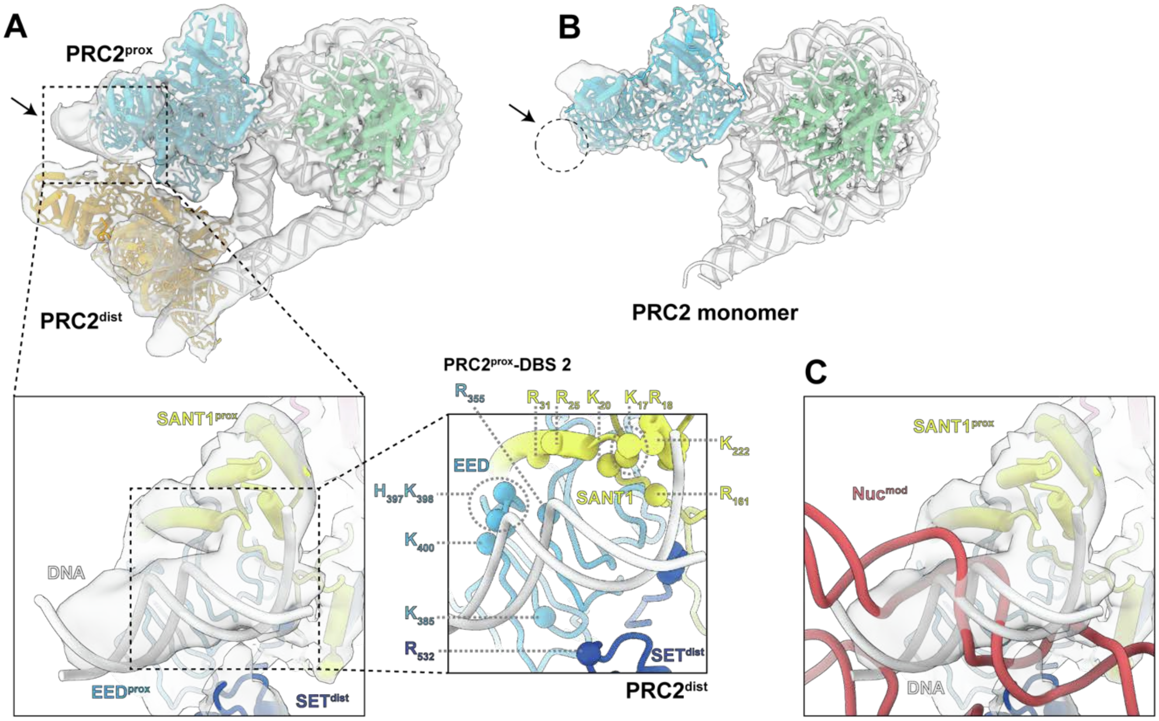

Supplementary Figure 5: Structural features of the PRC2 dimer, related to Figure 2.

(A) Additional density in the consensus map is indicated with arrows and suggested to be a short piece of dsDNA bound to DBS2 of PRC2^{prox}. Below: closeup of extra density with docked DNA model shows how positively charged residues on PRC2^{prox} and PRC2^{dist} could mediate binding. The binding surface would include portions of the PRC2^{prox} SANT1 domain which would only be positioned correctly when PRC2 is active, and the SBD helix is bent. (B) Comparison of (A) with the PRC2 monomer showing the absence of any additional density (arrow and circle). In the PRC2 monomer, the SBD helix is straight (Fig.4C) and the SANT1 domain is not available to mediate interaction with the additional density (C) DNA density and position with respect to an allosteric nucleosome (Nuc$_{mod}$) [8] showing that in the presence of an allosteric nucleosome the same DNA binding surface is used (DBS2).

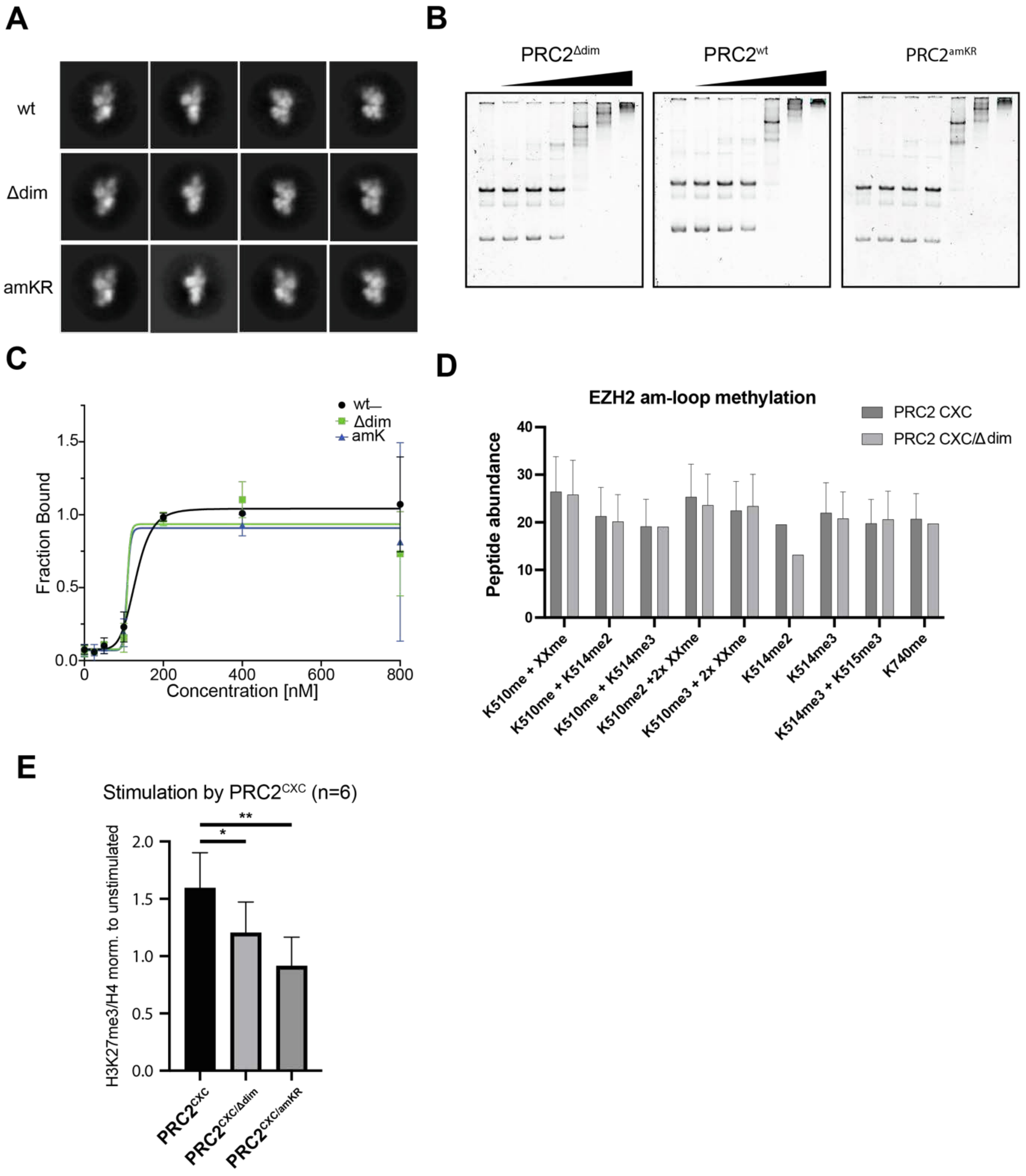

Supplementary Figure 6: Structure and activity of mutant PRC2 complexes, related to Figure 3.

(A) Representative 2D class averages of WT PRC, PRC2$^{\Delta dim}$ and PRC2^{amKR} obtained by negative-stain EM. Each row corresponds to the same view for all three complexes. (B) Nucleosome binding of WT PRC2, dimerization and automethylation mutants observed by EMSA. 50 nM mononucleosomes and two-fold titrations ranging from 25–800 nM PRC2. (C)

Densitometric quantification of n = 3-6 experiments as shown in B, based on the intensity of free nucleosomes. (D) Mass spectrometric analysis of the am-loop methylation states of recombinant PRC2^{CXC} and PRC2$^{CXC/\Delta dim}$. XXme = methylation was detected, but methylated K or R residues could not be assigned. (E) Quantitative analysis of HMTase activity assays as shown in Fig. 3, based on the band intensities of the H3K27me3 specific antibody relative to the H4 total histone loading controls. H3K27me3 intensities are shown normalized to the activity of unstimulated PRC2^{amKR}, i.e. in the absence of PRC2^{CXC}.

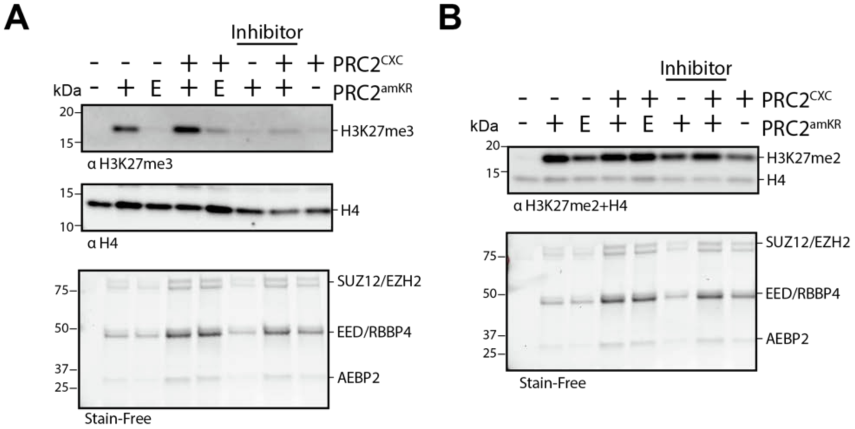

Supplementary Figure 7: HMTase activity assays with EED Y365A cage-mutant PRC2 and the EED inhibitor MAK683, related to Figure 3.

400 nM of PRC2^{CXC} was pre-incubated with SAM and subsequently incubated with 200 nM substrate nucleosomes as well as 200 nM PRC2^{amKR}. Allosteric activation via EED was disrupted by utilizing the EED Y365A cage mutant PRC2$^{amKR/EEDc}$ (lanes marked "E") or the EED inhibitor MAK638 (lanes marked "Inhibitor").

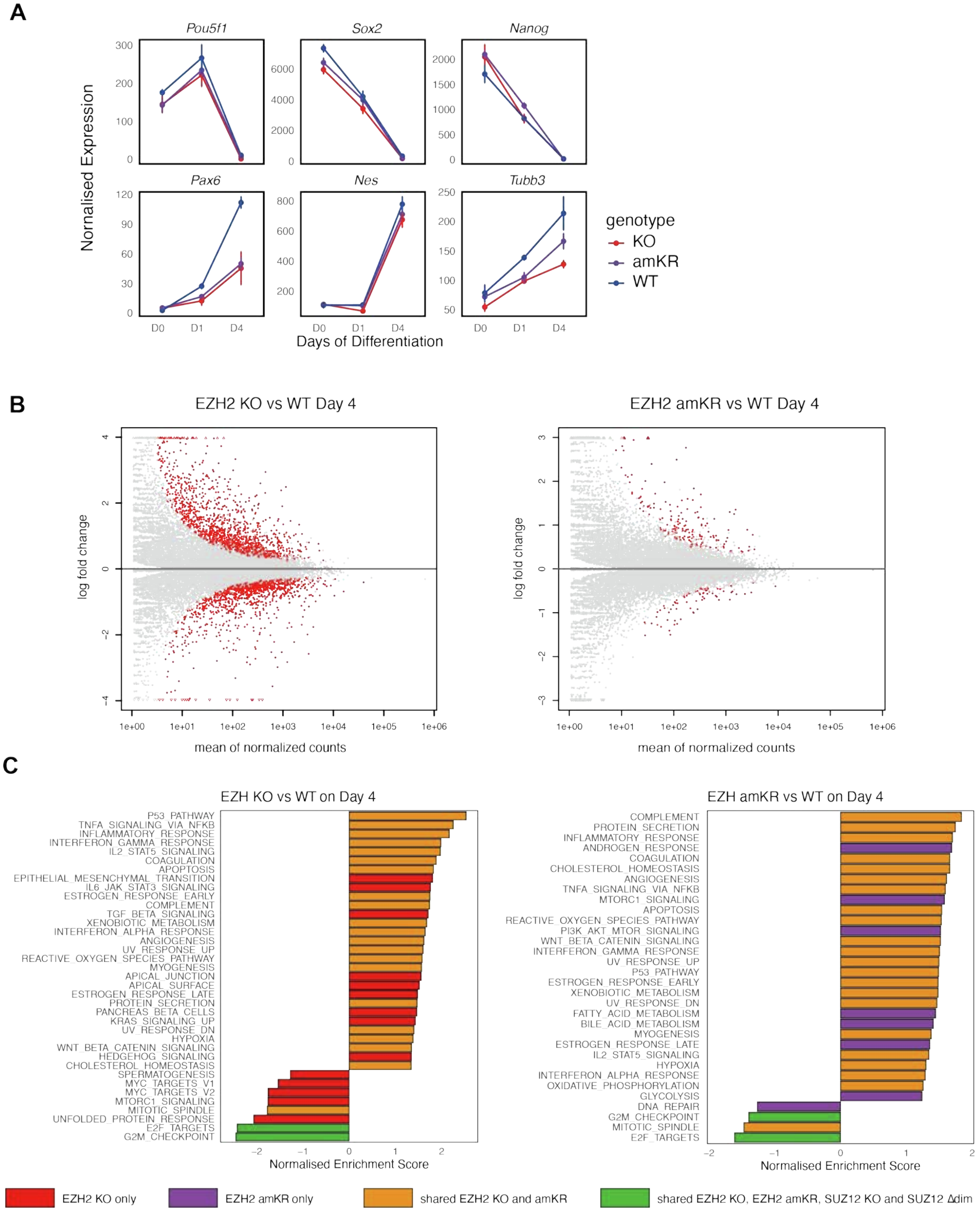

Supplementary Figure 8: Transcriptome analyses of EZH1/2 dKO, WT EZH2 and EZH2^{amKR} rescue mESCs, related to Figure 6.

(A) Gene expression profiles during RA-driven differentiation of pluripotency markers (upper panel) and neural trajectory markers (lower panel). Normalized expression data was derived from RNA-seq data and is shown as mean with standard deviation from three independent

biological replicates. Samples were taken at day 0 (representing ESCs), day 1, and day 4. Blue, red and purple graphs correspond to WT, KO and amKR, respectively. (B) MA Plots showing the relationship between average log2 fold change (y-axis) and average expression (x-axis) to assess Differentially Expressed Genes (DEGs) in EZH1/2 dKO (left panel) and EZH2^{amKR} (right panel) relative to WT EZH2 cells. Significant DEGs are highlighted in red. (C) Gene set enrichment analysis (GSEA) for differentially regulated transcripts in EZH1/2 dKO vs WT EZH2 rescue (left panel) or EZH2^{amKR} (right panel) vs. WT EZH2 rescue. Normalised enrichment scores plotted as bars for significant pathways (NOM p-value <0.1). Positive values correspond to gene sets enriched in EZH1/2 dKO or EZH2amKR rescue cells relative to the WT EZH2 rescue. Red, and purple bars correspond to differentially regulated pathways in dKO or EZH2^{amKR}, respectively. Orange bars highlight pathways consistently enriched between dKO and EZH2^{amKR} relative to WT EZH2 at day 4 of differentiation. Green bars highlight pathways consistently enriched between EZH1/2 dKO and EZH2^{amKR} relative to WT EZH2, as well as SUZ12 KO and SUZ12$^{\Delta dim}$ relative to WT SUZ12 at day 4 of differentiation.

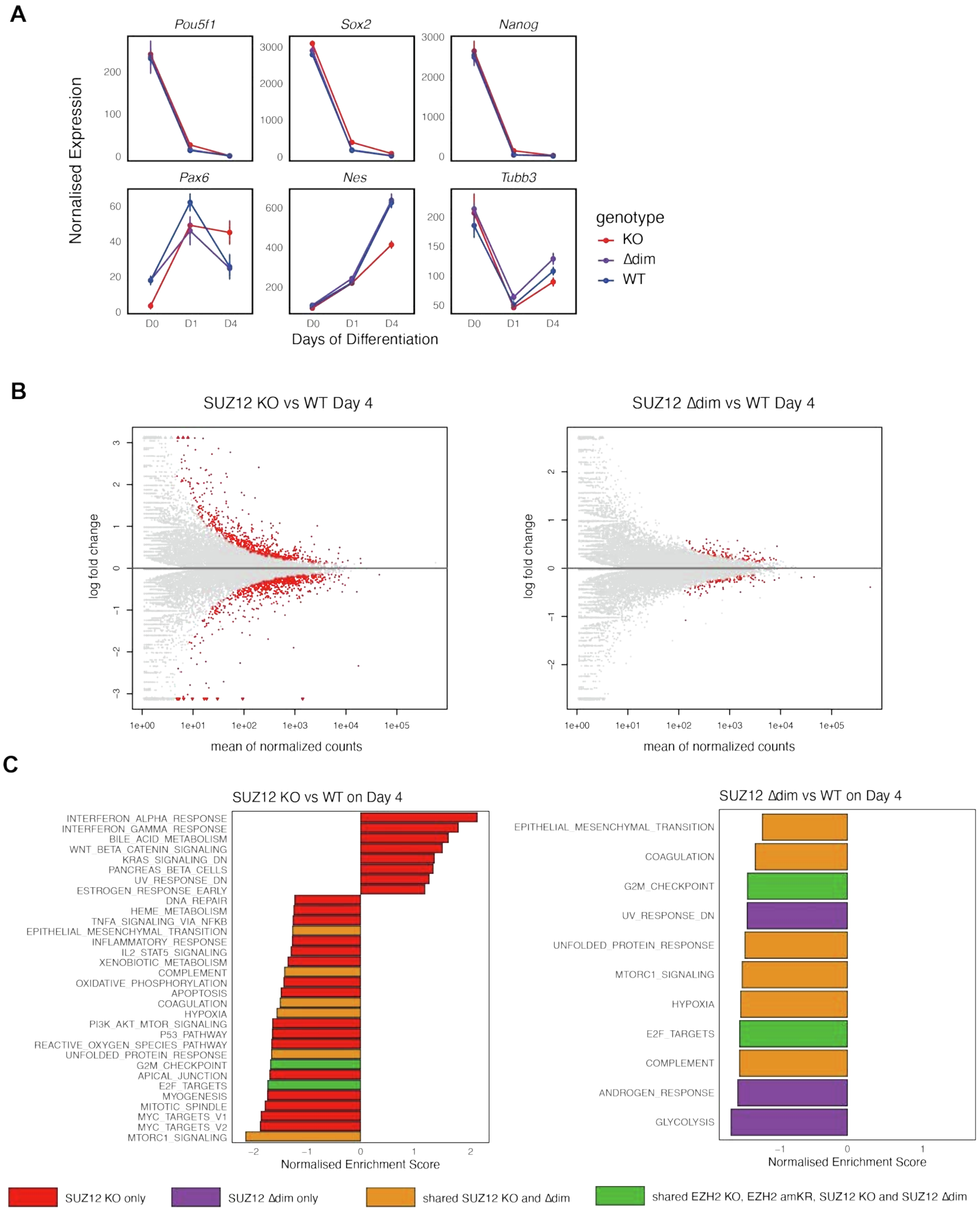

Supplementary Figure 9: Transcriptome analyses of SUZ12 KO, WT SUZ12 and SUZ12$^{\Delta dim}$ rescue mESCs, related to Figure 6.

(A) Gene expression profiles during RA-driven differentiation of pluripotency markers (upper panel) and neural trajectory markers (lower panel). Normalized expression data was derived from RNA-seq data and is shown as mean with standard deviation from three independent

biological replicates. Samples were taken at day 0 (representing ESCs), day 1, and day 4. Blue, red and purple graphs correspond to WT, KO and Δdim, respectively. (B) MA Plots showing the relationship between average log2 fold change (y-axis) and average expression (x-axis) to assess Differentially Expressed Genes (DEGs) in SUZ12 KO (left panel) and SUZ12$^{\Delta dim}$ (right panel) relative to WT EZH2 cells. Significant DEGs are highlighted in red. (C) Gene set enrichment analysis (GSEA) for differentially regulated transcripts in SUZ12 KO vs WT SUZ12 rescue (left panel) or SUZ12$^{\Delta dim}$ vs. WT SUZ12 rescue (right panel). Normalised enrichment scores plotted as bars for significant pathways (NOM p-value <0.1). Positive values correspond to gene sets enriched in SUZ12 KO or SUZ12$^{\Delta dim}$ rescue cells relative to the WT SUZ12 rescue. Red, and purple bars correspond to differentially regulated pathways in KO or EZH2^{amKR}, respectively. Orange bars highlight pathways consistently enriched between dKO and SUZ12$^{\Delta dim}$ relative to WT SUZ12 at day 4 of differentiation. Green bars highlight pathways consistently enriched between EZH1/2 dKO and EZH2^{amKR} relative to WT EZH2, as well as SUZ12 KO and SUZ12$^{\Delta dim}$ relative to WT SUZ12 at day 4 of differentiation.

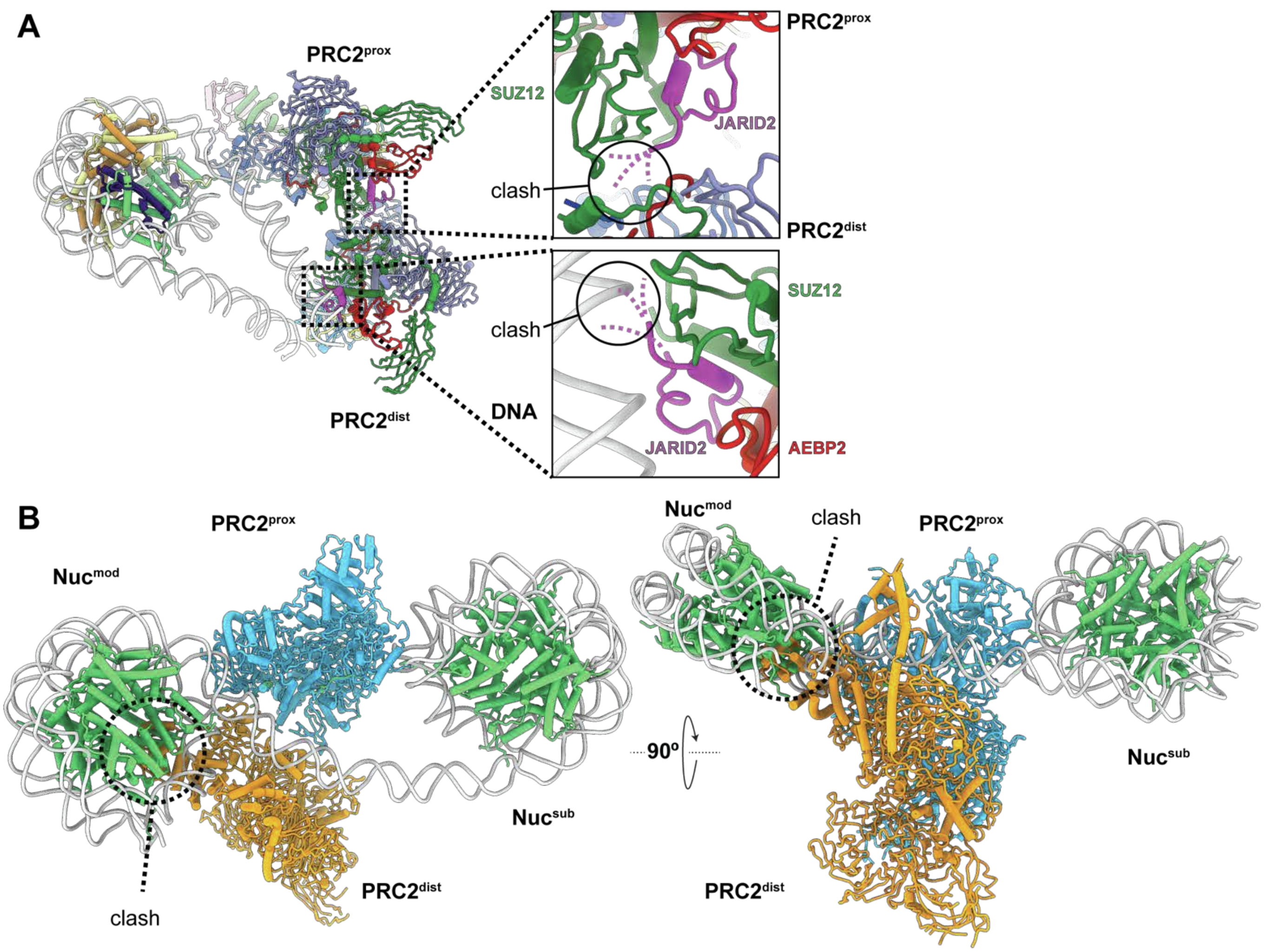

Supplementary Figure 10: Predicted steric incompatibility of the PRC2 dimer with JARID2 and the dinucleosome, related to Figure 7 and STAR methods.
(A) predicted incompatibility between JARID2 and the allosteric PRC2 dimer. The position of JARID2 was derived through alignment of each PRC2 complex with either PDB 6C23 or 6C24. Unmodeled JARID2 segments will likely block the SUZ12-SUZ12 dimer interface (top) and/or prevent SUZ12-DNA binding at DBS3 (bottom). (B) predicted clash between the allosteric PRC2 dimer and the PRC2-dinucleosome. The modified, allosterically activating nucleosome occupies the same space as the allosterically activating PRC2^{dist}.

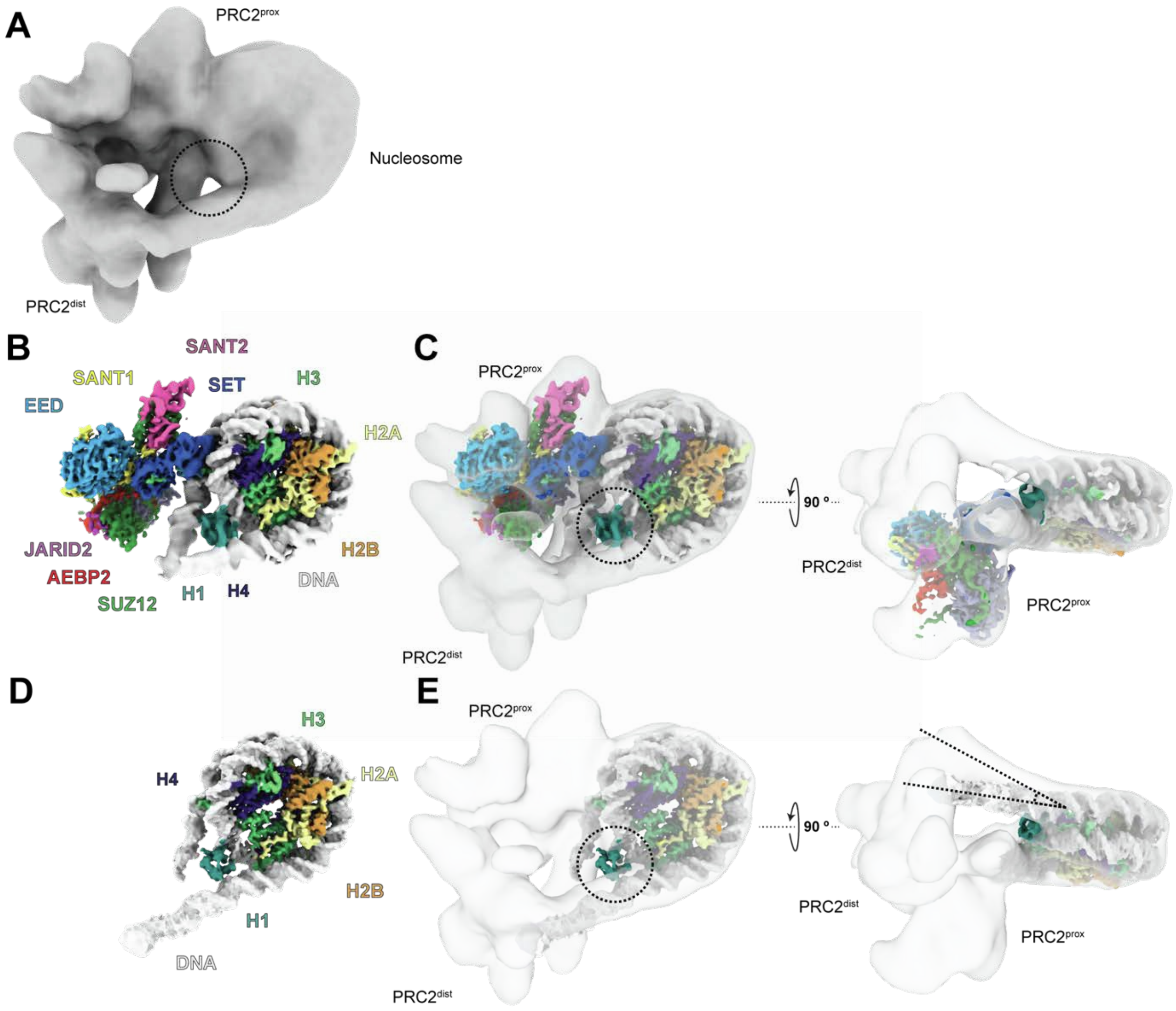

Supplementary Figure 11: The allosteric PRC2 dimer is incompatible with H1 binding, related to Figure 7 and STAR methods.

(A) Cryo-EM reconstruction of PRC2 dimer obtained in the presence of H1 in the sample. The circle indicates where H1 density would have been expected. (B) Cryo-EM reconstruction of PRC2 containing cofactor JARID2 bound to a nucleosome containing H1 (chromatosome) at 3.6 Å resolution. H1 (teal) is bound at the nucleosomal dyad, contacting the linker DNA. (C) Superposition of (A) and (B) shown in two orthogonal views. The circle shows the absence of H1 density in the allosteric dimer. (D) Cryo-EM reconstruction of the chromatosome in the absence of PRC2. (E) Superposition of (A) and (D) based on alignment of H1 in (D) and (C) shown in two orthogonal views. The right panel shows how the trajectory of the chromatosomal linker DNA is incompatible with the DNA geometry seen in the allosteric PRC2 dimer structure.

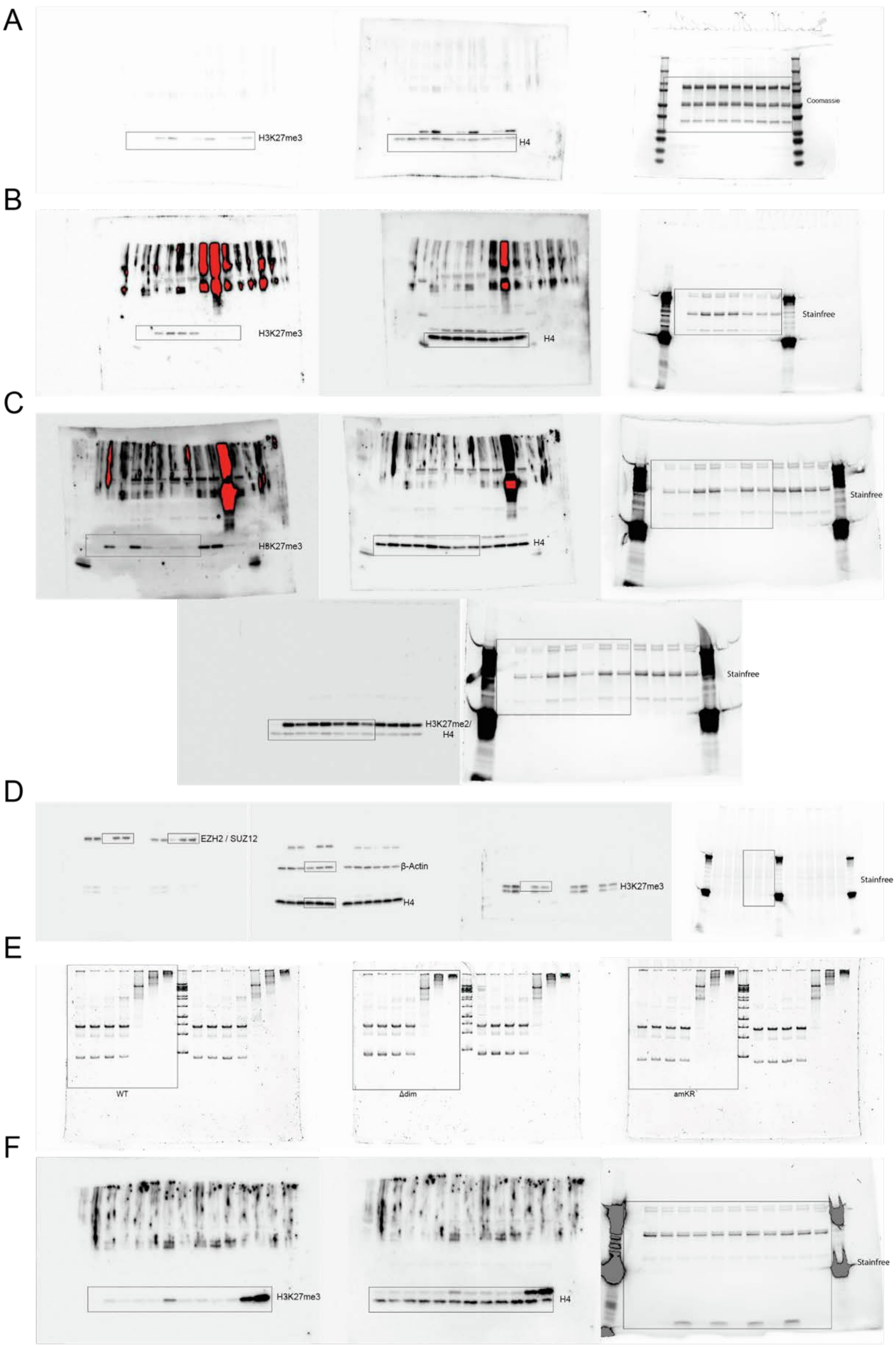

Supplementary Figure 12: Raw data for Western Blots and native gels, related to Figures 3, 4, 6, S6 and S7. *Uncropped images used (A) in Fig. 3C, (B) in Fig. 3D, (C) in Fig 4D, (D) Fig. 6A, (E) in Fig. S6B and (F) in Fig. S7A/B.*
